# New machine learning and physics-based scoring functions for drug discovery

**DOI:** 10.1038/s41598-021-82410-1

**Published:** 2021-02-04

**Authors:** Isabella A. Guedes, André M. S. Barreto, Diogo Marinho, Eduardo Krempser, Mélaine A. Kuenemann, Olivier Sperandio, Laurent E. Dardenne, Maria A. Miteva

**Affiliations:** 1 Laboratório Nacional de Computação Científica, Petrópolis, 25651-075 Brazil; 2 Fundação Oswaldo Cruz, Rio de Janeiro, 21040-361 Brazil; 3 Inserm U973, Université Paris Diderot, Paris, France; 4 Structural Bioinformatics Unit, CNRS UMR3528, Institut Pasteur, 75015 Paris, France; 5 Inserm U1268 “Medicinal Chemistry and Translational Research”, CiTCoM, UMR 8038, CNRS, Université de Paris, 75006 Paris, France

**Keywords:** Drug discovery, Computational biophysics, Cheminformatics

## Abstract

Scoring functions are essential for modern in silico drug discovery. However, the accurate prediction of binding affinity by scoring functions remains a challenging task. The performance of scoring functions is very heterogeneous across different target classes. Scoring functions based on precise physics-based descriptors better representing protein–ligand recognition process are strongly needed. We developed a set of new empirical scoring functions, named DockTScore, by explicitly accounting for physics-based terms combined with machine learning. Target-specific scoring functions were developed for two important drug targets, proteases and protein–protein interactions, representing an original class of molecules for drug discovery. Multiple linear regression (MLR), support vector machine and random forest algorithms were employed to derive general and target-specific scoring functions involving optimized MMFF94S force-field terms, solvation and lipophilic interactions terms, and an improved term accounting for ligand torsional entropy contribution to ligand binding. DockTScore scoring functions demonstrated to be competitive with the current best-evaluated scoring functions in terms of binding energy prediction and ranking on four DUD-E datasets and will be useful for in silico drug design for diverse proteins as well as for specific targets such as proteases and protein–protein interactions. Currently, the MLR DockTScore is available at www.dockthor.lncc.br.

## Introduction

Structure-based drug design and virtual screening have become common approaches for drug discovery. The predictive performance of scoring functions is essential for such methodologies^1–3^. However, accurate prediction of protein–ligand binding affinity remains a major challenge for current scoring functions. Despite the improvement over the last years of empirical, force-field or knowledge-based scoring functions, most of them still show unsatisfactory correlation with the experimental binding affinity or are based on meaningless description of protein–ligand interactions exhibiting overestimated accuracies in some cases^4–6^.

Empirical scoring functions are based on a set of individual contributions or interaction descriptors calibrated by regression or statistical approaches using a training set of experimental affinity data for protein–ligand complexes^7,8^. Improvement of scoring functions can be achieved by developing new terms, training on larger high-quality datasets or using sophisticated machine learning-based algorithms for regression analysis, e.g. XGBoost and LightGBM boosting approaches^9–13^. Next, solvation and entropy contributions are key for ligand binding^14–20^. Although several previous scoring functions have considered such effects^14,15,17,19^common limitations of scoring functions are related to often neglecting them^10,21–23^. New scoring functions based on more precise physics-based descriptors to better represent protein–ligand recognition process are thus needed. Furthermore, a number of studies demonstrated that scoring functions performance is very heterogeneous across different target classes^22–26^. Target-specific scoring functions have shown to achieve better affinity prediction performance than general scoring functions trained over diverse protein families^21–23,27–29^.

In this work, we developed a set of new empirical scoring functions, named DockTScore, to estimate protein–ligand binding affinity by explicitly accounting for physics-based interaction terms contributing to the binding free energy. Our models are based on the MMFF94S force field and trained and validated on high-quality large datasets properly curated. DockTScore scoring functions incorporate classical van der Waals and electrostatic energy terms, optimized terms accounting for solvation, lipophilic protein–ligand interactions and an improved estimation of ligand torsional entropy contribution to ligand binding for better describing of protein–ligand recognition. Firstly, we employed multiple linear regression (MLR)^30,31^ to ensure a physical interpretation of the individual term contribution. Then, we developed more sophisticated nonlinear scoring functions using support-vector machine (SVM) for regression (named “SMOReg”)^32^ and random forest (RF)^33^ algorithms using the theory-inspired physics-based terms selected from the initial MLR analysis. The development of scoring functions using physics-based descriptors representing protein–ligand recognition process together with the assessment of the accuracies of different linear and nonlinear models are important to avoid unrealistic overestimations of scoring functions accuracy due to some known biases, especially when training nonlinear models^4,6,34,35^.

In addition to general scoring functions appropriate for diverse protein targets, we have developed MLR, SMOReg and RF scoring functions for two specific protein classes: proteases, and protein–protein interactions (PPIs) to be targeted by small-molecule inhibitors (iPPIs). Proteases are key drug targets, for which focused scoring functions have already been developed (e.g. targets such as HIV-1 protease^35^). Interestingly, only one work has been reported thus far aiming at developing a linear scoring function to predict the binding affinity of inhibitors of PPIs^36^ using a training set of 27 PPIs complexes. Our MLR DockTScore for iPPIs gave new insights into the determinant factors contributing to inhibiting PPIs by small molecules. Moreover, we report here the first nonlinear scoring functions focusing on iPPIs and developed on 60 PPI complex structures carefully selected and curated. We evaluated the accuracy of affinity prediction and success of virtual screening to discriminate between active and decoys compounds of our scoring functions on four DUD-E datasets.

## Methods

### Data sets

#### Data sets of diverse protein–ligand complexes for general scoring functions

We trained and tested the general scoring functions appropriate for diverse protein targets based on the PDBbind v2013 refined set (http://www.pdbbind-cn.org/, version 2013), which is composed of 2959 protein–ligand complexes with binding affinity data manually collected from their original source^37–40^. PDBbind is known as the largest dataset of high-quality structures available for the development and validation of docking-scoring methods. The refined set was constructed according to several criteria concerning (i) the quality of the structures, (ii) the binding affinity data and (iii) the nature of the complex. Binding affinities in PDBbind comprise a large interval of values, ranging from 1.2 pM (1.2 × 10^−12^ M) to 10 mM (1.0 × 10^−3^ M). We converted the original binding constants to energy unit in kcal mol^−1^.

The PDBbind core set, a subset of the refined set widely used as benchmarking data for evaluation of docking-scoring methods, was used here to assess the performance of our general scoring functions as an external test set only, not being used during the training step. The core set version 2013 is composed of 195 protein–ligand complexes carefully collected from the refined set for comparative studies of scoring functions^38–40^.

#### Data sets for target-specific scoring functions

We selected a random subset from the PDBbind v2013 refined set according to specific ranges of the EC Number, (Enzyme Commission Number (EC Number) is a system of enzyme nomenclature that numerically classifies enzymes based on the chemical reaction catalyzed.) ranging from 3.4.11.0 to 3.4.25.69, to create a dataset for training and testing the scoring function focused for proteases, resulting in a subset composed of 783 structures (Table S1).

To create the dataset for inhibitors of protein–protein interactions (iPPIs), we took the X-Ray-based iPPIs dataset previously described in Kuenemann and colleagues^41^, which was composed of 85 protein–ligand complexes. Here, we collected the binding affinity data from the original sources and manually prepared each complex using the Protein Preparation Wizard from Maestro (Maestro, version 9.7, Schrödinger, LLC, New York, NY, 2014). From the initial 85 iPPIs dataset, 25 complexes were removed due to their low resolution (value higher than 2.5 Å), the presence of covalently bound ligands or absence of affinity data. The remaining 60 structures were suitable for training and testing the specific scoring functions for iPPIs (Table 1).

**Table 1:** The iPPIs dataset.

Protein (short name)	Total^a^	Affinities (kcal mol^−1^)	Training^b^	Test^c^
Bcl2-like/BAX	10	−12.636^d^, −5.244^e^	7	3
Bromodomain2/Histone	2	−9.968, −8.561	2	0
Bromodomain4/Histone	11	−9.931, −6.145	9	2
K-Ras/SOS1	1	−4.712	1	0
MDM2-like/P53	20	−12.768, −6.737	14	6
Menin	1	−10.404	0	1
Xiap/Smac	7	−11.278, −5.378	6	1
E1/E2	1	−10.051	1	0
IL2/IL2R	1	−6.910	1	0
LEDGF/Integrase	4	−10.490, −6.676	2	2
ZipA/ftsZ	2	−6.685, −5.544	2	0
Total	60		45	15

^a^Total number of protein–ligand complexes in the dataset.

^b^Number of complexes in the training set.

^c^Number of complexes in the random test set.

^d^Binding affinity of the strongest protein–ligand interactions.

^e^Binding affinity of the weakest protein–ligand complex.

#### Training and test sets

All datasets were randomly separated into a training set with 75% of the structures and an independent test set with the remaining 25% structures (Table S1). For the general scoring functions, the core set (N = 195) was extracted from the refined set, initially containing 2959 complexes. Thus, the random selection of complexes for the independent test and training sets was performed exclusively with the remaining 2764 complexes. The random 75% of the 2764 complexes used to train the general scoring functions is called “General::random” training set (N = 2073, Table S1. In addition, we tested the influence of the training data set size on the predictive capacity for the general scoring functions. Thus, we also trained general scoring functions using all the 2764 protein–ligand complexes (called here “General::all”, Table S1). In this case, the predictive performance was evaluated only on the v2013 core set (N = 195).

For proteases, the training set was composed of 587 complexes and the test set was composed of 196 distinct complexes, not being used during the training step. Given the smaller size of the iPPI dataset, we characterized the composition of both training and test sets according to the protein families and the range of the binding affinity data (Table 1). Complexes of MDM2-like/P53 interacting with small ligands are the most frequent with 20 available structures, followed by complexes of Bromodomain4/Histone (11 complexes) and Blc2-like/BAX (10 complexes).

#### Preparation of the structures

Protein–ligand complexes of the v2013 refined set consist of the complete unit taken from Protein Data Bank (PDB)^42^ (rcsb.org) and is available as prepared structures following an automatic procedure with some manual inspection performed by Li and colleagues^38^. Originally, the protein–ligand complexes were prepared following a simple protonation scheme considering a neutral pH: (i) all carboxylic acid and phosphate groups were deprotonated, and (ii) all aliphatic amine, guanidine and amidine groups were protonated. As well known, the correct assignment of both protein and ligand protonation/tautomeric states is crucial for correct binding mode and affinity predictions, but is a very time-consuming task for a large number of ligands^43–45^. In this work, we applied an improved protocol for the preparation of the structures of the v2013 refined set using the Protein Preparation Wizard from Maestro (Maestro, version 9.7, Schrödinger, LLC, New York, NY, 2014). Protonation assignment and hydrogen-bond optimization were performed using ProtAssign and PROPKA^46^ considering the presence of the bound ligand. Protonation and tautomeric states of the ligand were calculated using Epik^47^ (Epik, version 2.7, Schrödinger, LLC, New York, NY, 2014). Metal ions were considered as cofactors, and all waters were removed from the structures. Finally, energy minimization was performed to optimize the hydrogen atoms positions. A special attention was paid for the preparation of the core set due to its importance for the benchmarking studies. The protonation/tautomeric states of the binding-site residues and the bound ligand of the core set were further visually inspected and appropriate corrections were made guided by the original reference corresponding to the respective crystallographic structure and the Protoss program^48^. The curated core set (protein, ligand and cofactors) is freely available in the Supplementary Material. All structures of the iPPIs datasets and the proteases from DUD-E were prepared using the same protocol adopted for the core set.

### Physics-based interaction terms

In this work, we implemented and evaluated several physicochemical terms contributing to the binding free energy to obtain pertinent descriptors for the derivation of the empirical scoring functions: protein–ligand electrostatic interactions ($E_{coul}$), van der Waals interactions ($E_{vdW}$), lipophilic contact interactions ($E_{lipo}$), polar ($E_{polar\_solv}$) and nonpolar ($E_{np\_solv}$) solvation contributions, and ligand torsional entropy contribution ($E_{entropy}$).

#### Electrostatic and van der Waals protein–ligand interactions

The protein–ligand electrostatic and van der Waals interactions are calculated using the MMFF94S force field^49,50^. The MMFF94S force field was parameterized using high-quality ab initio quantum–mechanical data and demonstrated to accurately reproduce protein–ligand binding geometry in docking studies^51,52^. The electrostatic interaction $E_{coul}$ was calculated using:

$$E_{coul} = \frac{{332.0716q_{i} q_{j} }}{{\varepsilon \left( {R_{ij} + \delta_{elec} } \right)}}$$

where $q_{i}$ and $q_{j}$ are the partial charges of atoms *i* and *j*, $\varepsilon$ is the dielectric constant, $R_{ij}$ is the distance between the centers of the atoms *i* and *j*, and $\delta_{elec} = 0.05$ is the electrostatic buffering constant. The partial charges $q_{i}$ and $q_{j}$ are calculated through a bond-charge-increment method starting from an initial formal charge of the atom *i* ($q_{i}^{0}$) and adding the bond-charge-increment contributions ($\omega_{ki}$), which reflect the polarity of the covalent bonds of the atoms *i* and *k*:

$$q_{i} = q_{i}^{0} + \sum \omega_{ki}$$

In this work, we evaluated two sigmoidal distance-dependent dielectric functions to consider the electrostatic screening due to the dielectric medium of protein–ligand complexes. The first one developed by Hingerty and colleagues^53^ is currently implemented in the MMFF94S functional form used by the DockThor program for protein–ligand docking^51,52^ (available as a web server at https://www.dockthor.lncc.br):

$$\varepsilon \left( r \right) = 78 - 77\left( {\frac{r}{2.5}} \right)^{2} \frac{{e^{r/2.5} }}{{\left( {e^{r/2.5} - 1} \right)^{2} }}$$

where $r$ is the internuclear separation between the atoms *i* and *j*.

The second dielectric function was formulated by Ramstein and Lavery, allowing to change both the maximal value of the dielectric constant ($D$) and the limiting value of the dielectric ($D_{i}$) when the interatomic distance approaches 0 ($\varepsilon \left( r \right) \to D_{i}$ when $r \to 0$)^54^. Here, we tested $D_{i}$ values of 1 and 4 to simulate the relatively low dielectric at the interior of protein binding sites^55^.

$$\varepsilon \left( r \right) = D - \left( {\frac{{D - D_{i} }}{2}} \right)\left[ {\left( {rs} \right)^{2} + 2rs + 2} \right]e^{ - rs}$$

$r$ is the internuclear separation between the atoms *i* and *j*, $s = 0.16$ is the slope of the sigmoidal segment and $D = 78$.

The van der Waals potential ($E_{vdW}$) as implemented in the MMFF94S force field representing a “Buffered 14–7” form^50^ includes specific buffering constants $\delta_{vdW}$ and $\gamma = 0.12$:

$$E_{vdW} = \varepsilon_{ij} \left( {\frac{{\left( {1 + \delta_{vdW} } \right)R_{ij}^{*} }}{{R_{ij} + \delta_{vdW} R_{ij}^{*} }}} \right)^{7} \left( {\frac{{\left( {1 + \gamma } \right)R_{ij}^{*7} }}{{R_{ij}^{7} + \gamma R_{ij}^{*7} }} - 2} \right)$$

where $R_{ij}$ is the interatomic distance (Å), $\varepsilon_{ij}$ is the well depth (kcal mol^−1^) and $R_{ij}^{*}$ is the minimum-energy separation (Å), which depends on the MMFF94S types of the atoms *i* and *j*. The original buffering constant $\delta_{vdW} = 0.07$ was replaced in this work by $\delta_{vdW} = 0.67$, which was empirically obtained to produce a more softened version of the van der Waals potential noted as $E_{vdWS}$.

#### Lipophilic protein–ligand interactions

We developed two descriptors $E_{lipo}$ to calculate the lipophilic contact interactions effect $E_{lipo}$ by summing all hydrophobic atom pairs between the ligand and the protein following the previously proposed functional forms in ChemScore^56^ and X-Score^57^ scoring functions. For each of them, the atoms considered for lipophilic contacts were: (i) all carbon atoms, or (ii) any non-hydrogen atom with MMFF94S partial charge *q* in the interval $- 0.4 < q < + 0.4$. We empirically estimated this range of partial charges through analysis of several protein–ligand complexes parameterized with the MMFF94S force field. The $E_{lipo}$ descriptor for each lipophilic contact following e.g. the ChemScore is calculated by:

$$E_{lipo} = \begin{cases}1, &d\leq d_{vdW}+0.5{\AA}\\1-\frac{d-d_{vdW}+0.5}{3}, &d_{vdW}+0.5{\AA}<d\leq d_{vdW}+3.5{\AA}  \\ 0,&d>d_{vdW}+3.5{\AA}\end{cases}$$

where $d$ is the distance between the pairs of atoms and $d_{vdW}$ is the sum of their van der Waals radii.

#### Polar and nonpolar solvation contributions

In this work, the solvation contribution was calculated using a polar solvation term, which accounts for the loss of polar interactions of the charged groups of both protein and ligand with the solvent, and a nonpolar solvation term, which reflects the desolvation of the hydrophobic protein and ligand groups due to binding. The polar solvation term $E_{polar\_solv}$ was calculated by summing up the number of charged atoms becoming buried after the complex formation and not interacting with a charged atom in the protein–ligand complex. In this term, two charged atoms were considered as interacting if the distance between them ($d$) was equal to or lower than $d_{vdW} + 1.0 {\AA}$, where $d_{vdW}$ is the sum of their van der Waals radii. A charged atom was defined as a non-hydrogen and a non-carbon atom with a partial charge $\left| q \right| > 0.8$.

The nonpolar solvation $E_{np\_solv}$ was calculated based on the total loss of the solvent-accessible surface area (SAS) of the protein and the ligand due to the binding converted into energy ($E_{np\_solv}$ in kcal mol^−1^) following Kuhn and Kollman^58^. The SAS of atoms in the free and complexed states was calculated with the program MSMS^59^.

$$E_{np\_solv} = G_{np} complex - \left( {G_{np} protein + G_{np} ligand} \right)_{free}$$

where $G_{np}$ is calculated by:

$$G_{np} = 0.0092*SAS + 0.82$$

#### Ligand torsional entropy contribution

We revisited here the ligand torsional entropy term based on the conformational component of the ligand entropy and arising from the loss of the torsional degrees of freedom for a flexible ligand upon binding. Instead of a crude approximation based on the total number of all rotatable bonds^14–17,19^, we propose an improved estimation of the lost torsional freedom of the ligand by considering only the rotatable bonds, which become “frozen” due to binding. Similar approaches were previously adopted to approximate protein side-chain entropic contributions^15,60^.

The bonds are considered as “frozen” based on the change of the solvent-accessible surface areas of the ligand atoms directly involved in each rotatable bond, aiming to penalize only dihedrals that are unable to rotate after the complex formation.

**Figure 1:**
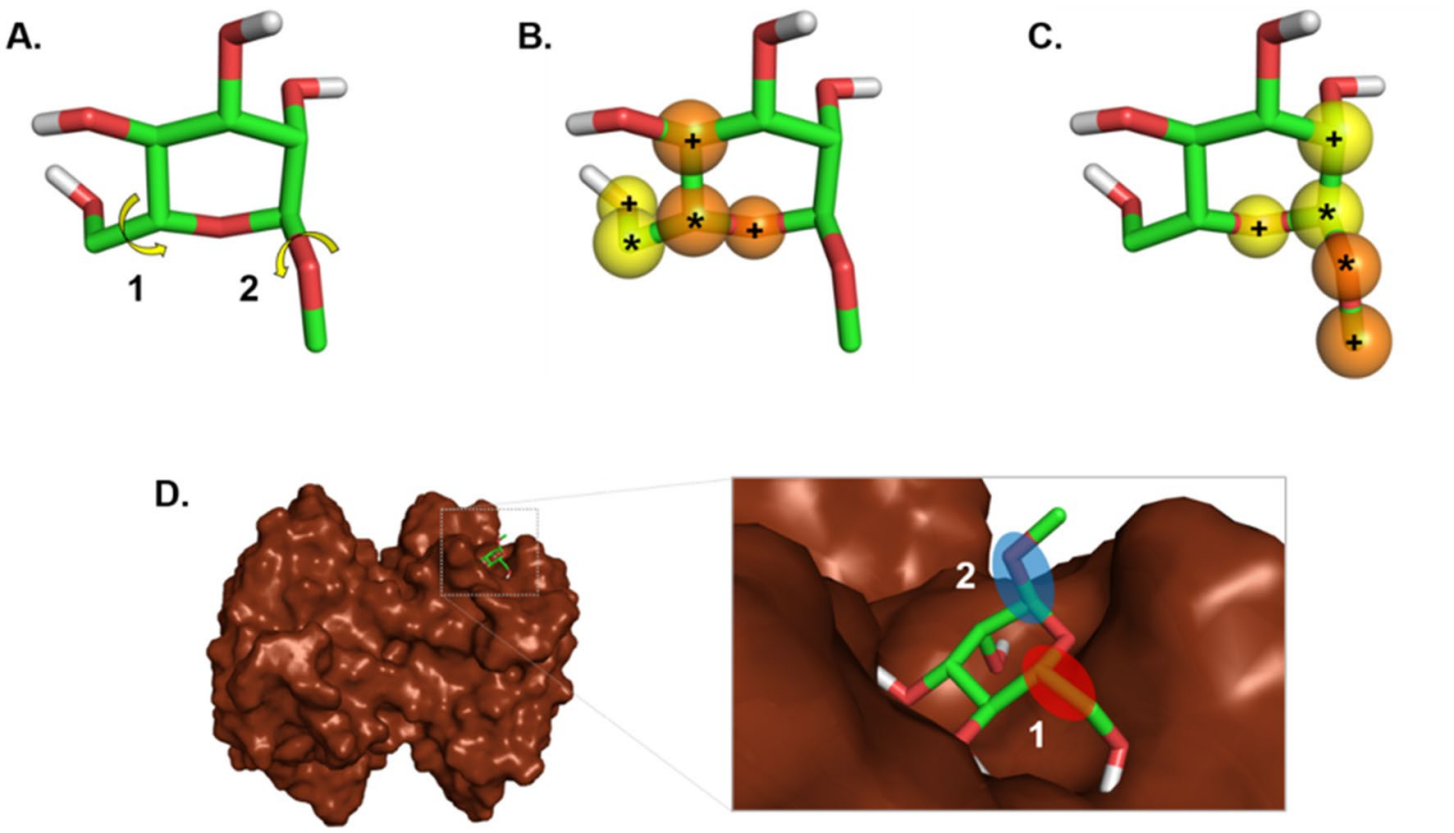
Illustration of the algorithm for computing the ligand torsional entropy term. (**A**) Selection of the rotatable bonds in the ligand. (**B** and **C**). Each rotatable bond is divided into two sides (i in yellow and j in orange) and the root (*) and the neighboring ( +) atoms are detected. (**D**) A rotatable bond is considered as frozen if both sides become buried with more than 50% due to the binding (case 1). If at least one side does not become buried with more than 50% due to the binding the rotatable bond is not taken into consideration (case 2).

Firstly, each rotatable bond of the ligand (Fig. 1A) is divided into two sides for the two atoms *i* and *j* (Fig. 1B,C). Each side is composed of (i) the atom *i*, which is directly involved in the bond (symbol *), and (ii) the first neighbors of the atom *i* (symbol +). The same procedure is applied to the other side (atom *j*). The change of the SAS (ΔSAS) for each side upon the binding is computed taking into account all atoms of the side. If SAS decreases ≥ 50% for the two sides, the rotatable bond is considered as frozen due to the binding. We consider that a hiding of a rotatable bond by more than 50% is significant for the ligand flexibility, and thus critical to the change of the ligand entropy due to the binding. In fact, the protein receptor is kept rigid during the docking and slight protein movements could compensate for a small change of the SAS of a ligand rotatable bond. We thus take into consideration only those bonds becoming frozen due to the binding for the ligand torsional entropy contribution estimation (Fig. 1D).

### Derivation of linear scoring functions

We performed the selection of the descriptors based on the assumption that the major contributions to the free energy of binding are the intermolecular interactions, represented by the van der Waals and electrostatic interactions between the protein and the ligand, and the solvation and entropy changes due to the binding. We developed thus independent descriptors accounting for van der Waals and electrostatic interactions, protein–ligand lipophilic contacts, the change of the conformational entropy of the ligand, and polar/nonpolar solvation contribution to the binding (see their definition in “Physics-Based Interaction Terms”). Then, we selected the best descriptors (see below), assuring that all above mentioned classes of interactions have been present in the final scoring functions, instead of using a combinatorial or sequential descriptors selection.

We applied multiple linear regression (MLR) ensuring a physical interpretation of the individual terms’ contributions. A tenfold cross-validation was used to select the best performing physics-based descriptors. This initial descriptor selection was applied only for the derivation of the general scoring function since it was trained with the largest training set containing diverse protein–ligand complexes. We started with the basic function F_MMFF_ containing the electrostatic term with the Ramstein dielectric function tending to 4, *Di* = 4, ($E_{coul4}$) and the soft van der Waals term ($E_{vdWS}$) based on the original MMFF94S force field. These two terms were selected since they achieved the best correlation among four combinations tested for the electrostatic and vdW terms (see Table S2).

Then, each of the remaining physics-based descriptors (lipophilic contacts, entropy, polar solvation and nonpolar solvation) was individually added to the basic function F_MMFF_ one at a time, to find the best variation for each of them leading to the best correlation on cross-validation experiments. Thus, the combinations evaluated herein were: F_MMFF_ + lipophilic contacts (4 variants), F_MMFF_ + entropy, F_MMFF_ + polar solvation, and F_MMFF_ + nonpolar solvation. The correlations obtained for all combinations are present in the Supplementary Material (Tables S3 and S4). We considered the best variation of each specific term to finally combine them into the general scoring function (F_final_ = F_MMFF_ + lipophilic contacts + ligand conformational entropy + polar solvation + nonpolar solvation). Next, the best combination of terms of the general scoring function was applied to the class-specific scoring functions, and was also used for the descriptors in the development of nonlinear scoring functions with machine learning methods.

### Derivation of nonlinear scoring functions

In this work, we also developed nonlinear scoring functions using the Support Vector Machine for Regression (SMOReg) and Random Forest (RF) algorithms. Such scoring functions were trained using the same physics-based descriptors selected for the final linear scoring functions.

Support Vector Machine (SVM) aims to find the hyperplane that maximizes the margin of separation between data classes. In particular, in the kernel application the original nonlinear separable data can be transformed to a linear hyperplane separable problem on a higher dimension space^61^. SMOReg uses the sequential minimal optimization (SMO) for training support-vector machines (SVM) models in regression problems. In regression problems, all prediction errors less than a value of ε are ignored (*insensitive-loss function*)^30,62^. This strategy reduces the risk of overfitting on the training set and is controlled by the complexity parameter *C*, which is user-defined together with ε.

Random Forests (RF) were introduced by Breiman in 2001 as a powerful strategy for ensemble learning^33^. The RF combines several random trees (*numTrees*) in a bagging ensemble model, often leading to excellent results in diverse classification problems^33,62^. The output variable of a RF model is usually an average value of the predictions of the regression trees (as used in this work), where the node splitting is performed using a finite subset of features randomly chosen (*numFeatures*).

All the machine-learning procedures were carried out using the Weka v3.8.3 package^30^. We explored diverse configurations of SMOReg and RF on a tenfold cross-validation procedure. For SMOReg, we varied the complexity parameter *C*, tolerance in loss function epsilon (ε), kernel (*puk* or *rbf*), gamma (γ) of the *rbf* kernel, and sigma (σ) and omega (ω) of the *puk* kernel. In the RF training, we explored the number of trees (*numTrees*) and the number of features that are randomly chosen for splitting the parent node (*numFeatures*).

The tested learning parameters and their optimal values found are present in Tables S5 and S6, respectively (see Supporting Information).

### Validation of the scoring functions

#### Binding affinity accuracy

The best model of each machine-learning algorithm was selected according to the Pearson’s Correlation Coefficient ($R$) using the tenfold cross-validation strategy. Then, we applied the scoring functions to the respective test sets to validate their affinity predictability according to *R* and root mean squared error (*RMSE*). Both *R* and *RMSE* were calculated using the experimental and predicted free energy of binding (ΔG_bind_):

$$R = \frac{{\mathop \sum \nolimits_{i = 1}^{N} \left( {y_{i} - \overline{y}} \right)\left( {t_{i} - \overline{t}} \right)}}{{\sqrt {\mathop \sum \nolimits_{i = 1}^{N} \left( {y_{i} - \overline{y}} \right)^{2} } \sqrt {\mathop \sum \nolimits_{i = 1}^{N} \left( {t_{i} - \overline{t}} \right)^{2} } }}$$

where $y_{i}$ and $t_{i}$ are respectively the predicted and the experimental binding affinities for the *i*-th complex, $\overline{y}$ and $\overline{t}$ are the arithmetic average values for *y* and *t* and *N* is the number of points in the data set.

$$RMSE = \sqrt {\frac{1}{N}\mathop \sum \limits_{i = 1}^{N} \left( {y_{i} - t_{i} } \right)^{2} }$$

where N is the number of points in the dataset, $y_{i}$ is the predicted binding affinity and $t_{i}$ is the experimental binding affinity.

#### Virtual screening experiments

In order to evaluate the success of our scoring functions to discriminate active and decoys compounds, we performed docking experiments using the protein–ligand docking program DockThor^51,52^ and re-scoring with DockTScore on core set and the DUD-E datasets^63^ for the proteases FA7 (coagulation factor VII, PDB code 1W7X), RENI (renin, PDB code 3G6Z), TRYB1 (tryptase β1, PDB code 2ZEC), and UROK (urokinase-type plasminogen activator, PDB code 1SQT), and the kinases AKT2 (serine/threonine-protein kinase AKT2, PDB code 3D0E), KIT (stem cell growth factor receptor, PDB code 3G0E) and MK01 (MAP kinase ERK2, PDB code 2OJG). Proteases were selected to evaluate the screening success of the DockTScore general and target-specific scoring functions trained on the PDBbind refined set due to the large size of the training set used to calibrate the focused scoring functions for proteases. The protease and kinase datasets from DUD-E were chosen according to the following criteria: (i) no metal ions interacting with the ligand, and (ii) co-crystallized ligand successfully redocked with the top-energy solution with RMSD ≤ 2.0 Å. For PPIs, we constructed screening datasets for Bcl2-like/BAX and MDM2/p53 systems composed of actives taken from the iPPI-DB^64^ database (https://ippidb.pasteur.fr/) and inactive compounds taken from the BDM chemical library available at ChemREST (https://chem-rest.pasteur.fr/#?&versioned_sources=8&used_filters =). The iPPI-DB is a database that contains the structure, some physicochemical characteristics, the pharmacological data and the profile of about 2000 modulators of protein–protein interactions. It contains exclusively small molecules and therefore no peptides. BDM compounds have been previously shown to be negative on MDM2 and Bcl2 interactions via fluorescence polarization assays^65^. For the PPIs screening datasets, we selected only the compounds without chiral centers and having only one protonation/tautomer state as predicted by Epik. Following the DUD-E sets construction, we selected randomly 50 inactives for each active compound to keep an adequate balance between actives and inactives to evaluate the scoring functions performance on virtual screening experiments. The PDB codes 3QKD and 4IPF were used for the receptor structures of the Bcl-2-like protein 1 and MDM2, respectively.

The docking poses were generated with the program DockThor for protein–ligand docking freely available as a web server at https://dockthor.lncc.br). The DockThor program uses a grid box to define the search space, the DMRTS genetic algorithm as the search algorithm, and an MMFF94S-based scoring function for pose prediction^51,52^. Configuration of the search space of each protein target was automatically determined according to the reference ligand: (i) the center of coordinates was defined as the center of coordinates of the ligand, (ii) the grid size was defined as the largest axis value of the ligand plus a tolerance of 6 Å on each dimension, (iii) the discretization (*i.e.* spacing between two points of the grid) was set to the default value of 0.25 Å except for the cases where the grid size was greater than 26 Å. The parameters of the search algorithm were set as follows for redocking experiments: (i) 24 docking runs, (ii) 1,000,000 evaluations on each docking run, (iii) initial population of 1,000 individuals. The MMFF94S-based scoring function for ranking the docking poses (*E*_*total*_) consists of (i) the torsional, electrostatic and Buf-14–7 van der Waals potential terms for the internal energy, and (ii) the electrostatic and Buf-14–7 van der Waals potential terms for the intermolecular interactions. The docking poses are clustered using our in-house tool *dtstatistic* using a criterion of diversity equals to 2.0 Å.

The screening experiments were performed using the computational facilities provided by the Brazilian SINAPAD (*Sistema Nacional de Alto Desempenho*, https://www.lncc.br/sinapad/) high-performance platform and the Supercomputer SDumont. We used a set of GA parameters named “virtual screening” for the screening experiments used to reduce the computational cost, consisting of 12 docking runs, 500,000 GA evaluations and initial population of 750 individuals. The top-energy docking pose ranked by the total energy *E*_*total*_ were selected for the virtual screening experiments and binding affinity predictions.

The screening success was evaluated according to the area under the curve for the receiver operation characteristics (ROC AUC), the enrichment factor at 1% of the screened libraries (*i.e.,* EF_1%_), and the Boltzmann-enhanced discrimination of ROC values (α = 20 and α = 100, respectively BEDROC20 and BEDROC100)^66^ using the open-source tool for virtual screening analysis Rocker^67^.

## Results

### Performance of physics-based terms for the scoring functions

The best correlation between the predicted and experimental affinities (R = 0.493) using tenfold cross-validation on the General::random training set (N = 2073) with MLR for a scoring function accounting only for $E_{vdW}$ and $E_{coul}$ was obtained with our softened version of the Buf-14-7 van der Waals potential ($E_{vdWS}$, with $\delta_{vdW} = 0.67$) and the electrostatic term using the sigmoidal dielectric function of Ramstein and Lavery^58^ with $D_{i} = 4$ (Table S2), noted here as $E_{coul4}$. The scoring function composed of only $E_{vdWS}$ and $E_{coul4}$ terms is noted in this work as the “basic scoring function” $F_{MMFF}$. No correlation was obtained in cross-validation experiments (R = 0.053) using only the two original MMFF94S force field terms $E_{vdW}$ Buf-14–7 (with $\delta_{vdW} = 0.07$) and $E_{coul}$ ($D_{i} = 1$). It is interesting to note that the best correlation was obtained with the softened version $E_{vdWS}$ , which is expected because no energy minimization of the complex structures was performed. Soft vdW potentials are more permissive for small clashes that can be present, in particular in structures generated by molecular docking without subsequent energy minimization. For X-ray derived structures shorter non-bonded atom–atom distances may be present when compared to energy minimized structures through classical force fields optimizations. Indeed, when dealing with non-optimized structures such as those used in X-ray models, it is indicated to softening the Buf-14-7 potential increasing the $\delta_{vdW}$ buffering constant^50^. The $E_{lipo}$ lipophilic contact term provided better results when nonpolar atoms were defined based on the MMFF94S partial charges instead of considering only carbon atoms, achieving here a Pearson correlation of R = 0.538 when added to the $F_{MMFF}$ basic scoring function (Table S3). This result indicated that our description of the atom types according to their partial atomic charges, specific for the MMFF94S force field is relevant. Adding our original and simple term for the polar solvation also improved the accuracy of the basic scoring function *F*_*MMFF*_ (R = 0.514). Similarly, adding the nonpolar solvation term to *F*_*MMFF*_ improved the correlation in tenfold cross-validation experiments (R = 0.503). In the same line, our proposed improved term for ligand torsional entropy contribution demonstrated to be important for the affinity prediction when associated with the basic scoring function, improving its correlation on cross-validation experiments (R = 0.507). The observed improvement due to our individual physics–based terms permitted their validation for further training of the general and target-specific empirical scoring functions.

### General scoring functions

The MLR coefficients obtained for the general scoring functions considering all validated six terms are shown in (Table 2). As expected, the coefficients are in accordance with the physical meaning of the corresponding terms (*i.e.,* favorable or unfavorable contribution). Energy terms such as van der Waals, electrostatic and nonpolar solvation increase the binding affinity when the associated coefficients have positive values and the corresponding interactions for $E_{coul}$ and $E_{np\_solv}$ are favorable for the binding. The empirical term related to the counting of the lipophilic atom pairs has a favorable contribution as the associated coefficient has a negative value. The polar solvation and the entropy terms are unfavorable as the coefficients are positive.

**Table 2:** Coefficients of the terms obtained for the general scoring functions trained with MLR.

Scoring functions	$E_{coul4}$	$E_{vdWS}$	$E_{lipo}$	$E_{entropy}$	$E_{polar\_solv}$	$E_{np\_solv}$	*c*_*0*_
*General::random*^a^	0.0039	0.0386	−0.0111	0.0560	0.1025	0.0169	−5.5197
*General::all*^b^	0.0045	0.0343	−0.0104	0.0605	0.0987	0.1180	−5.5178

^a^Scoring function trained with the random training set (N = 2073).

^b^Scoring function trained with the refined set minus core set (N = 2764).

MLR general scoring function trained with the random training set (N = 2073) exhibited a good performance on tenfold cross-validation experiments (R = 0.548) and on the curated core set (R = 0.602), and a lower performance on the random test set (R = 0.494) (Table S7). Our MLR general scoring function has predictive capacity comparable to the best evaluated linear scoring functions, with performance close to X-Score:HMScore (R = 0.614) and X = Score::SAS (R = 0.606) reported in the v2013 core set benchmark paper^39^.

**Figure 2:**
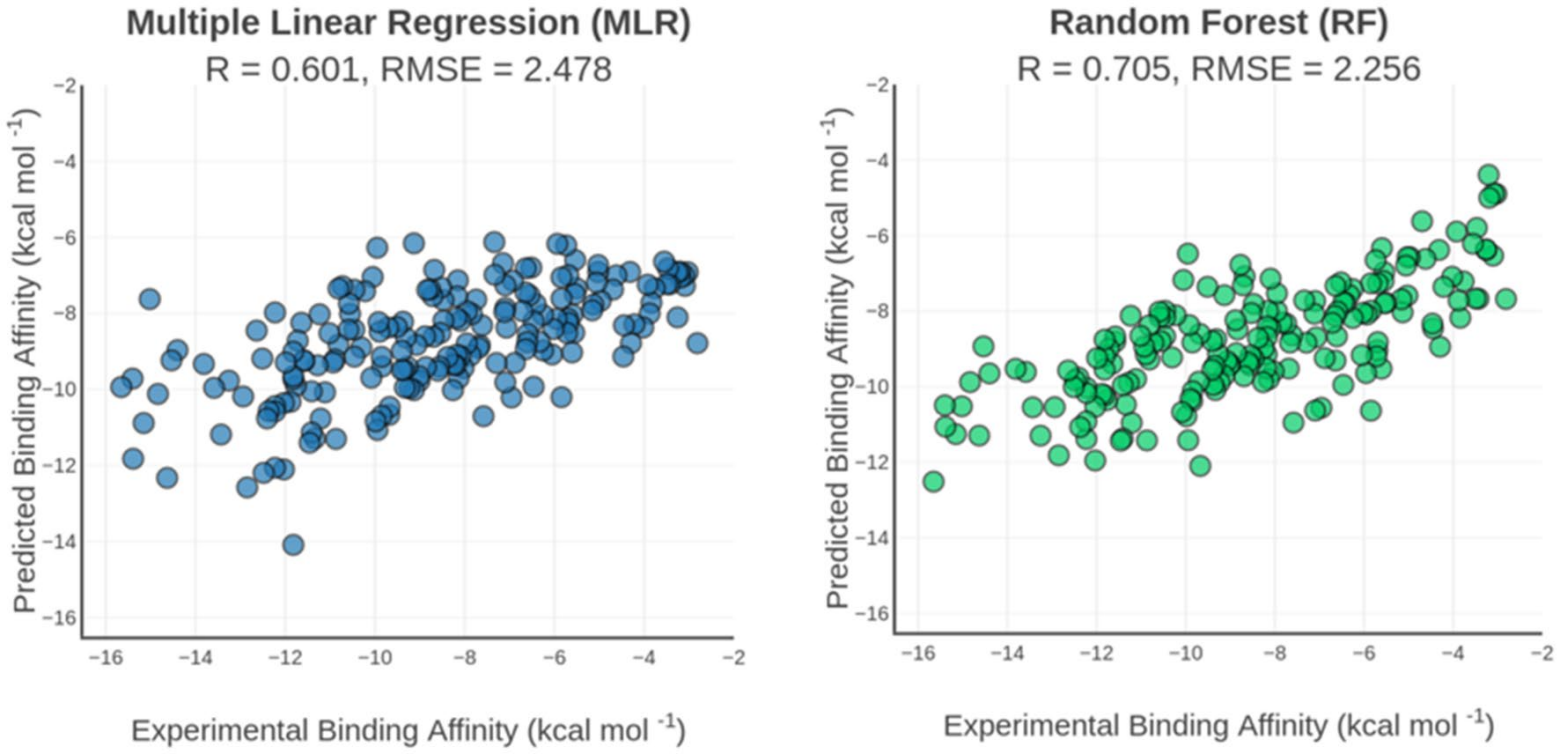
Correlation plot of the experimental and predicted binding affinities by the MLR (left) and RF (right) general scoring functions. Models trained on the PDBbind v2013 refined set (N = 2764) and evaluated on curated v2013 core set (N = 195). R is the Pearson’s correlation coefficient and RMSE is the root mean squared error given in kcal mol^−1^.

According to the tenfold cross-validation in the random general training set (N = 2073), it is seen that the SMOReg and RF models outperformed the MLR model, providing significantly better performances with R = 0.653 and R = 0.655, respectively (Table S7). These results confirm previous findings that nonlinear regression may better predict binding affinities than MLR and that the additive assumption adopted in the linear scoring functions could be too restrictive^68^. Using two different size training sets, the *General::all* one (N = 2764) and the *General::random* one (N = 2073) did not change the predictive performance of MLR model (R = 0.601 vs R = 0.602) while the larger training set improved the predictive performance of the SMOReg and RF models on the core set (Fig. 2 and Table S7), respectively R_SMOReg_ = 0.668 vs R_SMOReg_ = 0.687 and R_RF_ = 0.678 vs R_RF_ = 0.705. These results are consistent with other studies evaluating the influence of the training size, indicating that nonlinear scoring functions increase performance when more data is included in the training set while linear models seem to be less sensitive to the training set size^69,70^.

### Target-specific scoring functions

#### Proteases

**Figure 3:**
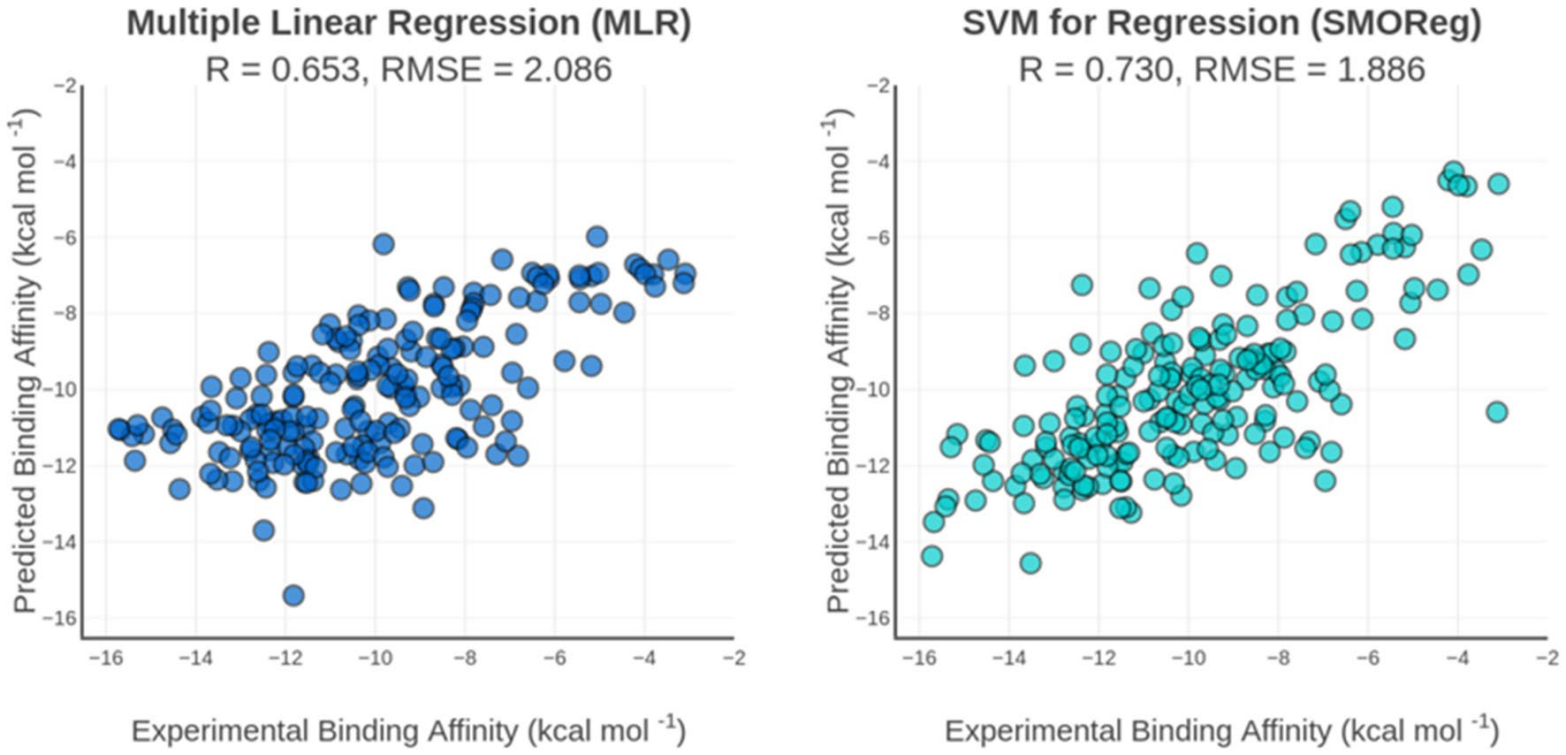
Correlation plot of experimental and predicted binding affinities by MLR (left) and SMOReg (right) specific scoring functions for proteases. The scoring functions were evaluated on the independent test set for proteases (N = 196). R is the Pearson’s correlation coefficient and RMSE is the root mean squared error given in kcal mol^−1^.

The linear scoring function for proteases exhibited good performance on the cross-validation experiments (R = 0.614) and on the independent test set (R = 0.653) (Fig. 3). All coefficients were very similar to those obtained for the general scoring function and their signals were in accordance with the physical meaning of the corresponding terms (Table 3). Likewise to the results observed for general scoring function, the nonlinear models for proteases exhibited significant improvements in the prediction capacity for both tenfold cross-validation experiment (R_SMOReg_ = 0.749 and R_RF_ = 0.735) and the independent test set (R_SMOReg_ = 0.730 and R_RF_ = 0.723).

**Table 3:** Coefficients of the terms obtained for the protease-specific scoring functions trained with MLR.

Scoring functions	$E_{coul4}$	$E_{vdWS}$	$E_{lipo}$	$E_{entropy}$	$E_{polar\_solv}$	$E_{np\_solv}$	c0
*Proteases*	0.0089	0.0399	−0.1120	0.0153	0.0515	0.0809	−4.8954

#### Protein–protein interactions (PPI)

For the iPPI linear scoring function, the representation of solvation as two independent terms leads to an unexpected favorable contribution of polar solvation instead of penalizing the buried charged atoms not involved in charge-charge interactions (Table 4). Thus, we decided to consider a single term for both polar and nonpolar solvation (called “oneSolv”), which has the same functional form of the nonpolar term but taking into account all heavy atoms, i.e., both polar and nonpolar ones. The solvation term “oneSolv” performed slightly better for the PPI-specific scoring function on cross-validation than using two solvation terms (R = 0.552 versus R = 0.545). Comparing the magnitude of the coefficients in the “oneSolv” model, the entropic and electrostatic terms exhibited a significantly higher contribution for iPPIs (Table 4). It has been widely demonstrated that iPPIs have higher hydrophobicity, aromaticity and molecular weight compared to enzyme inhibitors, as usually interacting within flatter, larger and more hydrophobic binding sites than the enzyme catalytic sites^41,71,72^. Given this, it is expected that the hydrophobic effect due to the binding represented here by the lipophilic contact and “oneSolv” solvation terms exhibit a strongly favorable contribution for this class of complexes. The unfavorable contribution of the $E_{vdWS}$ term might be due to some overlapping with the lipophilic contact and the “oneSolv” solvation terms. Further, a larger dataset set would allow to better evaluate the solvation contribution for inhibiting PPI.

**Table 4:** Coefficients of the terms obtained for the iPPI-specific scoring functions trained with MLR.

Scoring functions	$E_{coul4}$	$E_{vdWS}$	$E_{lipo}$	$E_{entropy}$	$E_{polar\_solv}$	$E_{np\_solv}$	*c*_*0*_
iPPIs	0.0505	0.0024	−0.0130	0.1967	−0.1698	1.0569	−0.7898
iPPIs-oneSolv	0.0335	−0.0207	−0.0153	0.2038	1.1227		−1.1397

Regarding the ligand entropy, it is clearly unfavorable for the binding. We expect that our improved entropic term penalizing only frozen rotatable bonds instead of all rotatable bonds is particularly important for the PPI class taken into account the large size of iPPIs and thus a possibly larger number of rotatable bonds. To confirm this hypothesis, we evaluated the linear scoring function for iPPIs on tenfold cross-validation experiments using the commonly used total number of rotatable bonds instead of the number of frozen torsions, and we obtained a slightly reduced correlation (R = 0.515). In this context, our entropic term demonstrated to be more appropriate for iPPIs than the total number of rotatable bonds.

**Figure 4:**
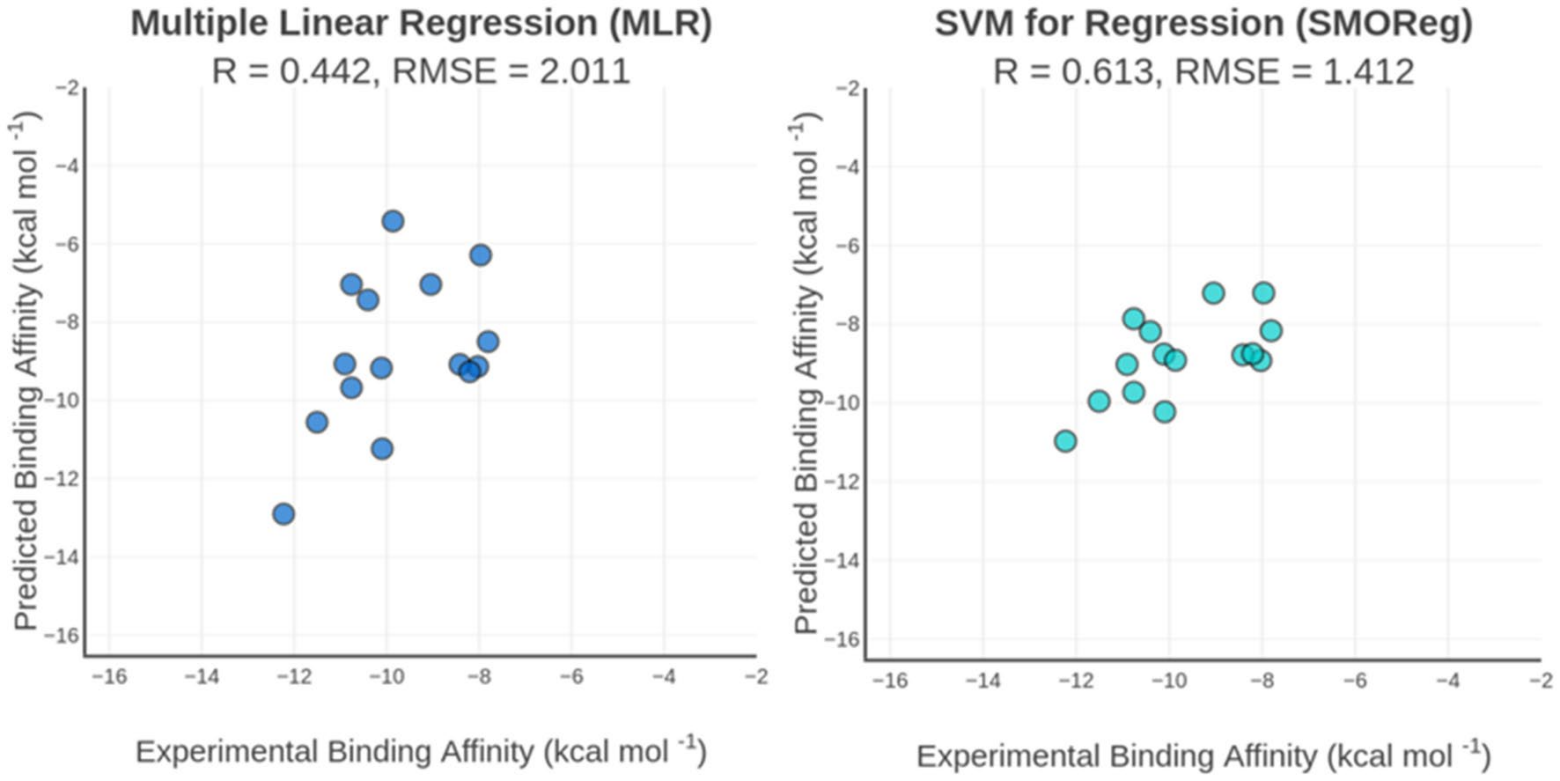
Correlation plot of predicted and predicted binding affinity by MLR (left) and SMOReg (right) specific scoring functions for iPPIs using one solvation term evaluated on the independent test set for iPPIs (N = 15). R is the Pearson’s correlation coefficient and RMSE is the root mean squared error given in kcal mol^-1^.

As expected, the nonlinear scoring functions specific for iPPIs, mainly the SMOReg model, improved the predictive performance when compared with the MLR model (Fig. 4), obtaining correlations of R_SMOReg_ = 0.600 and R_RF_ = 0.666 on the tenfold cross-validation, and R_SMOReg_ = 0.613 and R_RF_ = 0.478 on the test set. Curiously, despite the RF performing better on the tenfold cross-validation, the SMOReg model achieved a real improvement on the test set.

### Virtual screening

**Figure 5:**
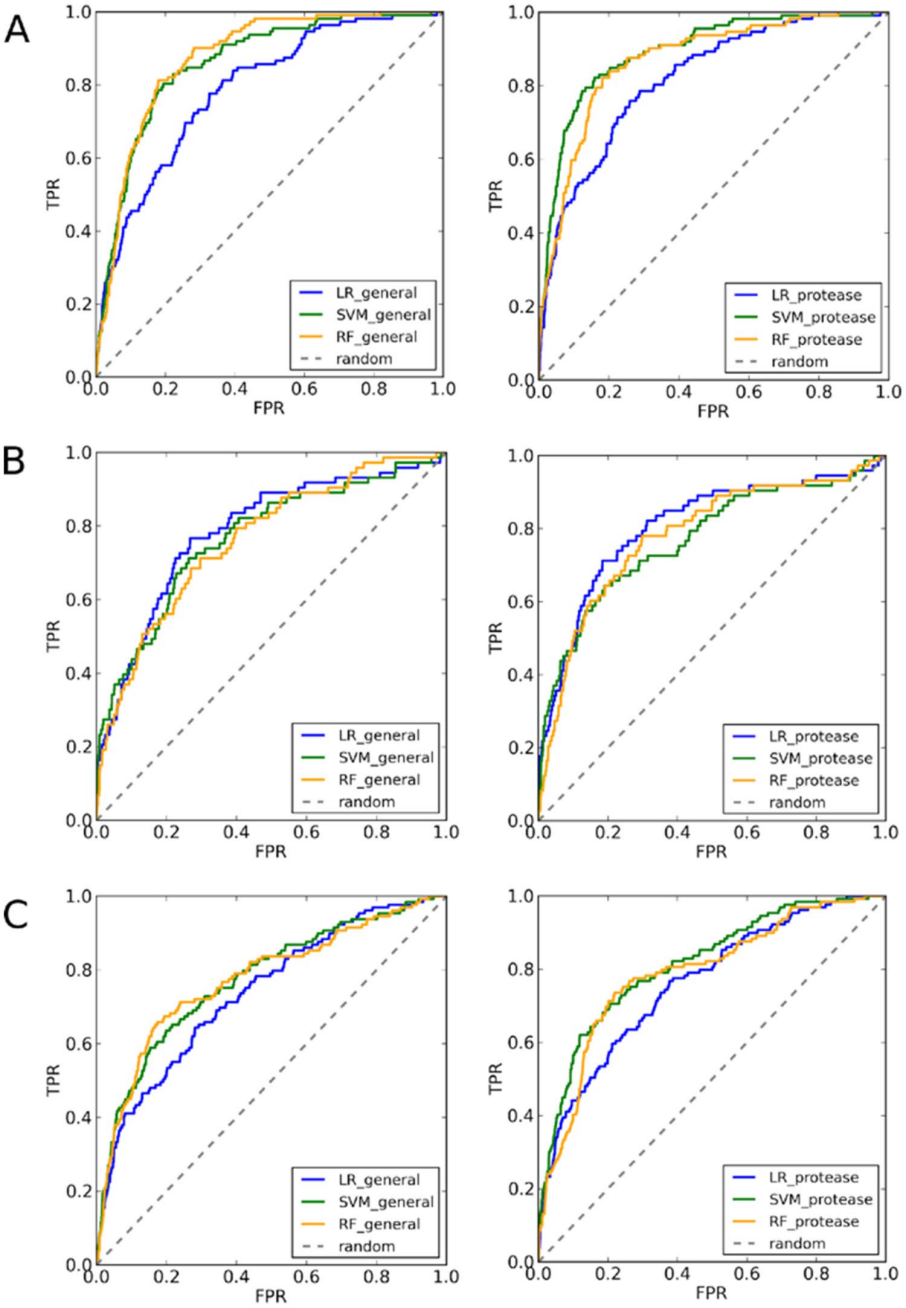
AUC ROC curves of the general (left) and protease-specific scoring functions (right) trained with MLR, SMOreg and RF for the FA7 (**A**), RENI (**B**), and UROK (**C**) datasets from DUD-E.

**Figure 6:**
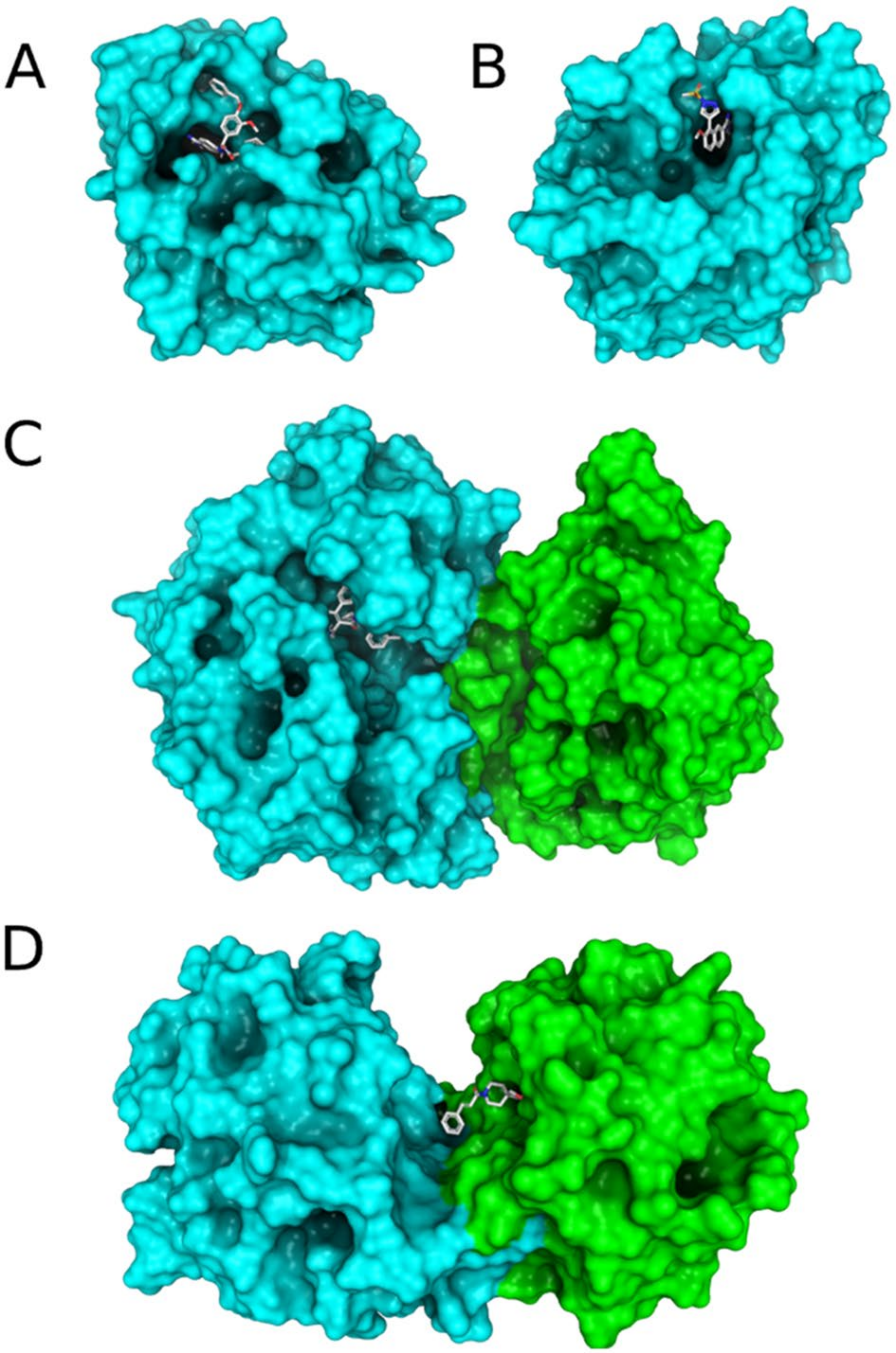
Surface representation of the binding sites of the proteases (**A**) FA7, (**B**) UROK, (**C**) RENI, and (**D**) TRYB1 colored by chain. The co-crystallized ligand is represented as sticks.

**Figure 7:**
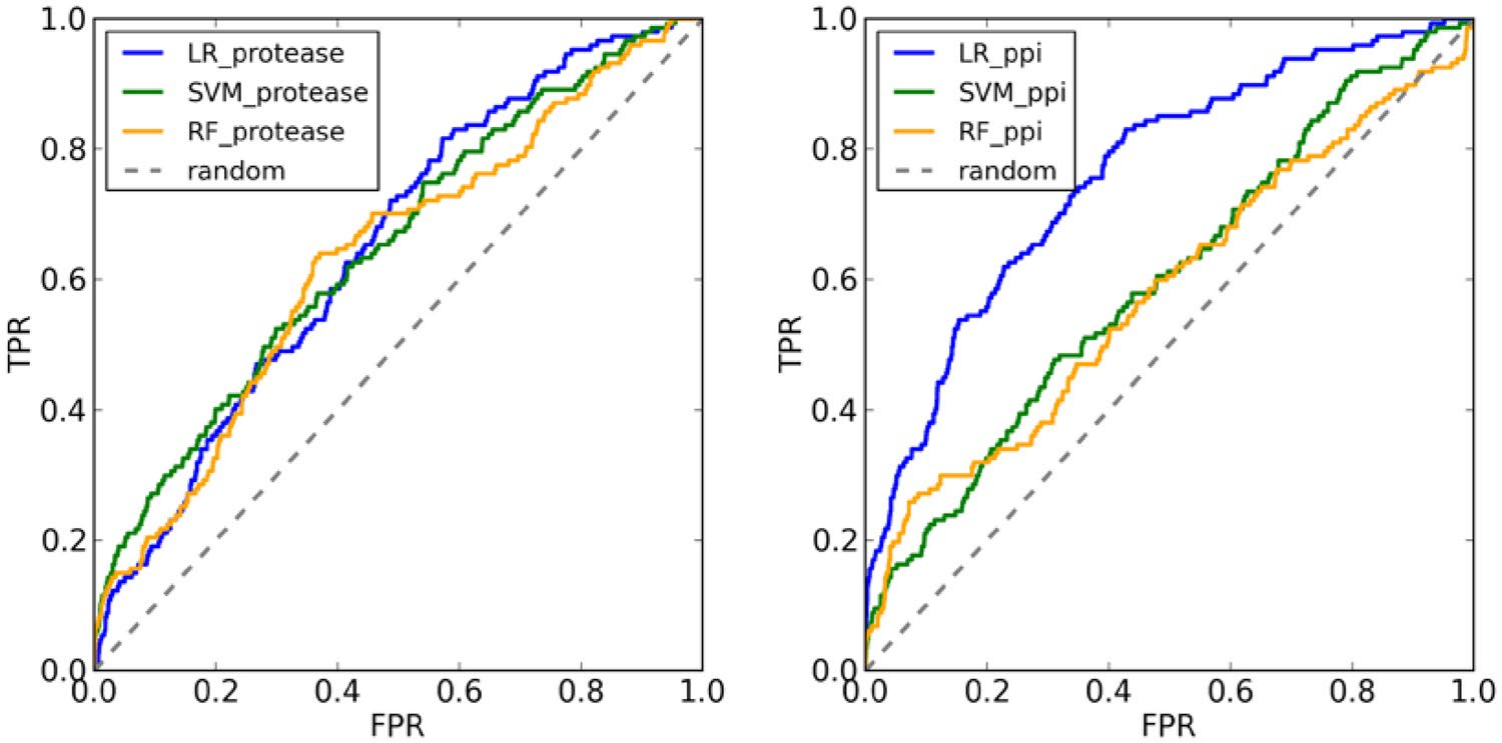
AUC ROC curves of the protease-specific (left) and PPI-specific (right) scoring functions trained with MLR, SMOreg and RF for the TRYB1 datasets from DUD-E.

In general, the DockTScore functions performed well in virtual screening experiments for the proteases (Table 5 and Fig. 5). According to the results, the best models achieved AUC ROC values better than 0.70 in most of the cases, while the early recognition of active compounds according to the EF_1%_ and the BEDROC values was variable between the different proteases studied, keeping in mind that BEDROC100 is very exigent for the early recognition of actives. Following the same trend observed for the binding affinity prediction*,* the nonlinear models generally performed better than the MLR models in terms of the screening success. Best results were obtained when using the specific scoring functions for proteases with the SMOreg model being the best-performing scoring function to distinguish actives from decoys. As an exception, the general and target-specific scoring functions exhibited low predictive performance for the TRYB1 target, with AUC ROC values lower than 0.651, a maximum EF1% only of 8, BEDROC20 of 0.203, BEDROC100 of 0.167. In this case, the accuracy is very low, taking into consideration that depending on the library size, often one can screen experimentally about 1% of the in silico screened compounds. The TRYB1 is a particular case, its binding site is remarkably exposed to the solvent. It is located in the interface of the two TRYB1 monomers belonging to the active tetramer^8^ sharing thus PPI-like properties. The co-crystallized ligand is bound with only one “frozen” rotatable bond in the dimer out of four rotatable bonds (Fig. 6). Therefore, we also evaluated the performance of the DockTScore PPI-specific scoring functions on the TRYB1 target (Fig. 7). Interestingly, the PPI-specific MLR scoring function outperformed the other scoring functions evaluated (*i.e.,* general and protease-specific, linear and nonlinear), achieving an AUC ROC curve of 0.762 (SMOreg_protease_ was 0.651), EF_1%_ = 15.626 (SMOreg_protease_ was 7.473), BEDROC20 = 0.291 (SMOreg_protease_ was 0.203) and BEDROC100 = 0.272 (SMOreg_protease_ was 0.167).

**Table 5:** Screening success of the general and target-specific scoring functions trained with MLR, SMOreg and RF for the FA7, RENI, TRYB1 and UROK datasets from DUD-E. *ac*, *dec* and *tot* are the number of active, decoy compounds and the total number of molecules in the final dataset (i.e., compounds that were docked and rescored with DockThor and DockTScore, respectively). Only the top-scored protonation state of a compound according to each scoring function (SF) was kept.

Target	Metrics	General SFs			Protease-specific SFs		
		MLR	SMOreg	RF	MLR	SMOreg	RF
FA7	AUC	0.789	0.860	0.875	0.818	0.893	0.869
*ac* = 112	EF1% (max = 52.973)	8.979	9.876	8.979	12.570	17.059	17.059
*dec* = 5,821	BEDROC20	0.299	0.346	0.328	0.350	0.478	0.397
*tot* = 5,933	BEDROC100	0.181	0.181	0.165	0.230	0.333	0.310
RENI	AUC	0.786	0.769	0.763	0.807	0.771	0.782
*ac* = 73	EF1% (max = 86.425)	16.462	20.577	10.975	17.834	16.462	8.231
*dec* = 6,236	BEDROC20	0.300	0.334	0.271	0.349	0.346	0.268
*tot* = 6,309	BEDROC100	0.253	0.281	0.155	0.283	0.207	0.119
TRYB1	AUC	0.619	0.649	0.614	0.651	0.651	0.633
*ac* = 147	EF1% (max = 51.633)	1.359	1.359	2.038	4.076	7.473	8.153
*dec* = 7,443	BEDROC20	0.099	0.103	0.080	0.141	0.203	0.169
*tot* = 7,590	BEDROC100	0.037	0.040	0.046	0.080	0.167	0.167
UROK	AUC	0.740	0.774	0.775	0.762	0.814	0.788
*ac* = 129	EF1% (max = 69.837)	7.760	8.536	6.208	11.640	14.743	10.088
*dec* = 8,880	BEDROC20	0.262	0.306	0.295	0.295	0.352	0.283
*tot* = 9,009	BEDROC100	0.123	0.147	0.118	0.179	0.232	0.182

**Figure 8:**
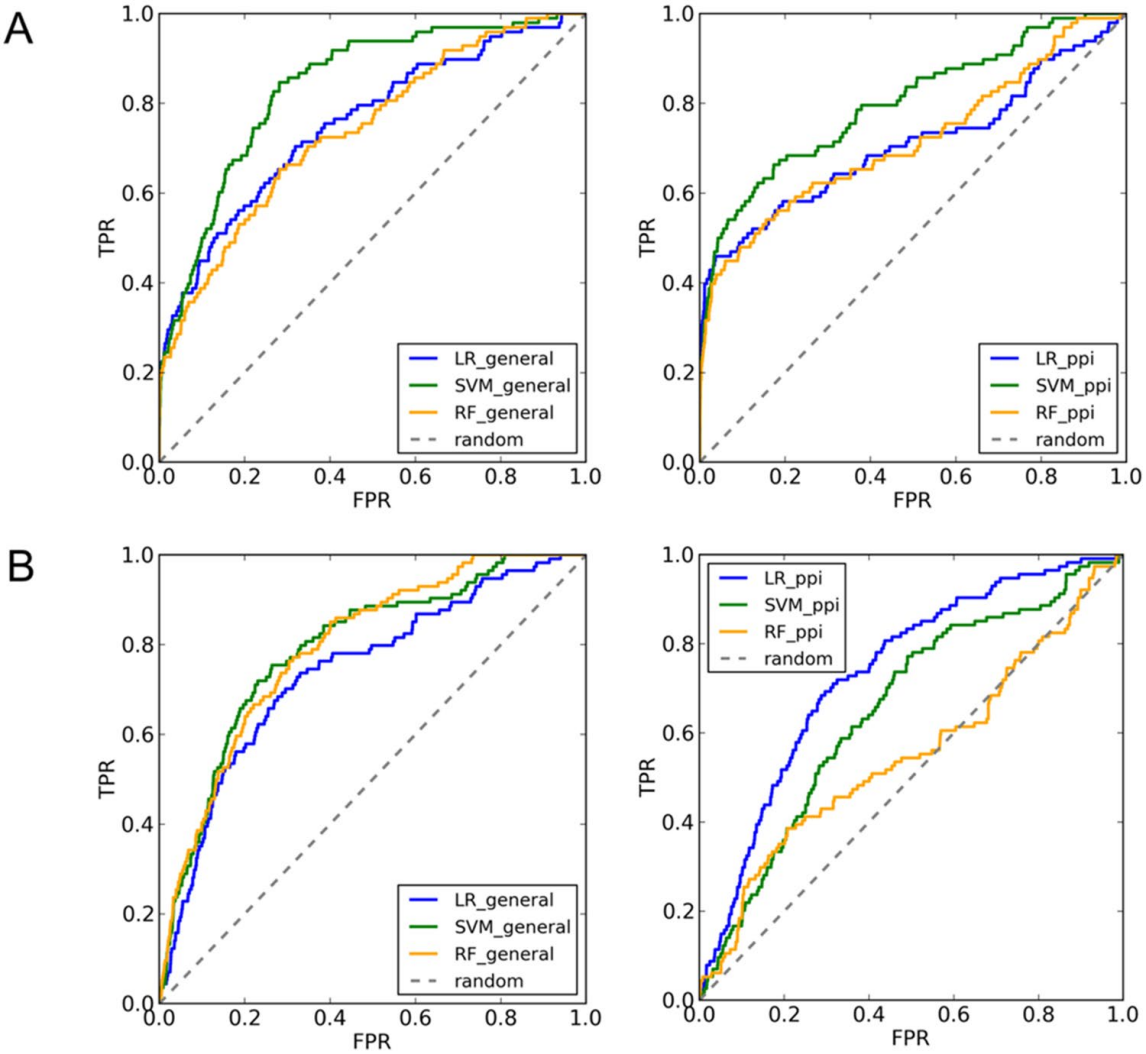
AUC ROC curves of the general (left) and PPI-specific (right) scoring functions trained with MLR, SMOreg and RF for the Bcl2-like/BAX (**A**) and MDM2/p53 (**B**) datasets.

The screening of actives and inactives on the two PPIs datasets resulted in AUC values better than 0.70 for the two targets for almost all scoring functions (Table 6 and Fig. 8), while the early recognition problem was successfully addressed only for the Bcl2-like system, reaching high BEDROC values of 0.474 (α = 20) for SMOreg and 0.539 (α = 100) for MLR. For the Bcl2-like protein/BAX system, the SMOreg scoring functions generally outperformed the other machine learning methods, whereas the PPI-specific scoring functions improved the EF_1%_ and BEDROC for all algorithms. Interestingly, the linear PPI-specific scoring function, with a satisfactory AUC ROC value of 0.709, obtained the best EF_1%_ value and the highest BEDROC100 value of 0.539. In the case of MDM2 target, the nonlinear general scoring functions outperformed the specific models in terms of AUC ROC, whereas the RF-based achieved the best overall screening performance. However, for this target all methods exhibited insufficient early recognition capacity according to the EF_1%_ and the BEDROC values.

**Table 6:** Screening success of the general and PPI-specific scoring functions trained with MLR, SMOreg and RF evaluated on the Bcl2-like protein/BAX and MDM2/p53 datasets. *ac*, *inac* and *tot* are the number of active, inactive compounds and the total number of molecules in the final dataset (*i.e.,* compounds that were docked and rescored with DockThor and DockTScore, respectively). Only the top-scored protonation state of each compound according to each scoring function (SF) was kept.

Target	Metrics	General SFs			PPI-specific SFs		
		MLR	SMOreg	RF	MLR	SMOreg	RF
Bcl2-like protein/BAX	AUC	0.755	0.838	0.740	0.709	0.801	0.716
*ac* = 98	EF1% (max = 51.510)	22.664	20.604	20.604	29.876	23.695	22.664
*inac* = 4,950	BEDROC20	0.370	0.375	0.330	0.471	0.474	0.418
*tot* = 5,048	BEDROC100	0.386	0.368	0.378	0.539	0.445	0.430
MDM2/p53	AUC	0.741	0.791	0.794	0.736	0.654	0.553
*ac* = 114	EF1% (max = 50.991)	4.400	4.400	6.154	2.637	1.758	5.275
*inac* = 5,699	BEDROC20	0.204	0.251	0.262	0.163	0.114	0.117
*tot* = 5,813	BEDROC100	0.010	0.112	0.124	0.068	0.042	0.090

**Figure 9:**
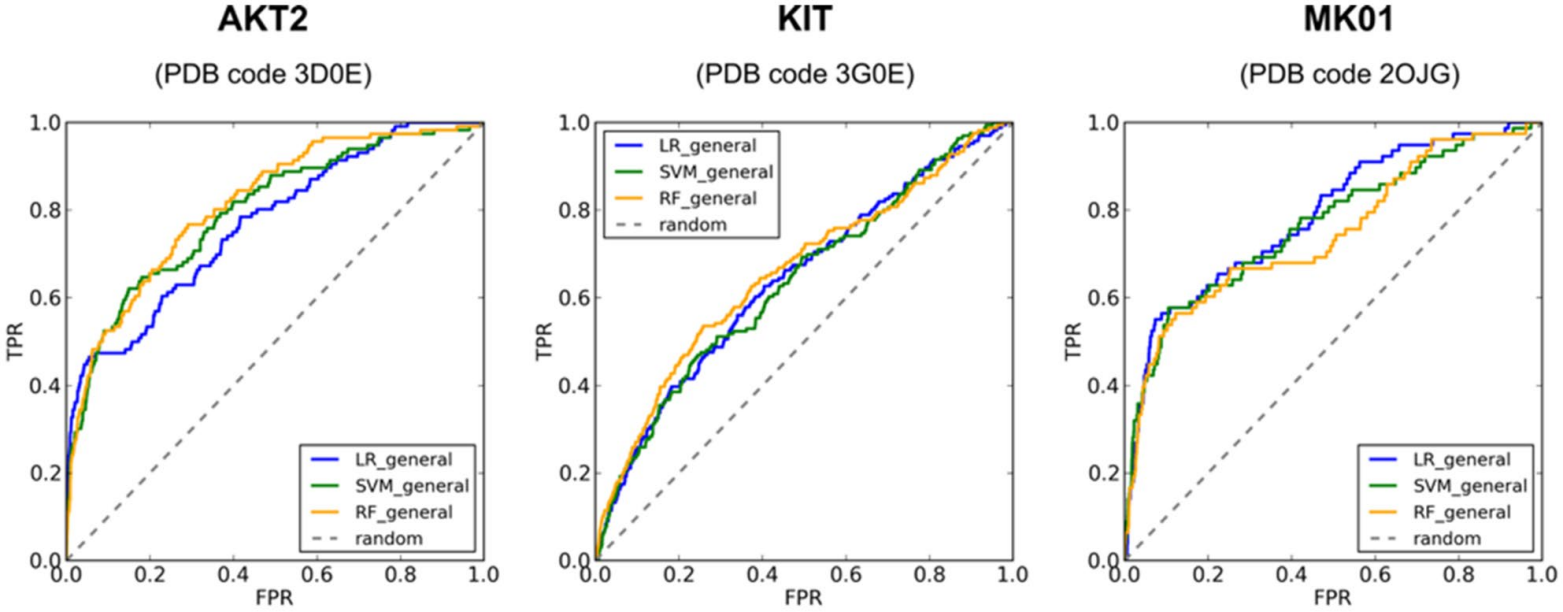
AUC ROC curves for the general scoring functions trained with MLR, SMOreg and RF evaluated on the AKT2, KIT and MK01 kinase datasets from DUD-E.

In addition to the proteases and PPIs targets, we also evaluated the performance of our general scoring functions trained with MLR, SMOreg and RF on three protein kinases datasets taken from DUD-E. Kinases are considered as challenging targets mainly due to binding site flexibility, which frequently leads to induced-fit effects due to ligand binding. Although DockTScore is not developed to deal with the receptor flexibility, our scoring functions exhibited satisfactory performances for two out of three kinases in virtual screening experiments, with AUC ROC values higher than 0.745 (Table 7 and Fig. 9). It is interesting to note that for AKT2 and MK01 targets, the MLR function showed better values for early the recognition metrics (e.g., EF, BEDROC20 and BEDROC100) than the SMOreg (AKT2and MK01) and RF (only for MK01) nonlinear functions. However, for the KIT target all the functions achieved insufficient performance for all evaluated metrics. It is important to note that in the screening experiments, we used a softened version of the MMFF94S Buf-14-7 force field to implicitly account for the protein flexibility to some extend explicitly permitting small clashes by reducing the repulsive energy between the protein–ligand atoms. However, the use of strategies that account for large movements of the binding site, such as ensemble docking with more than one representative structure of the protein, might be necessary to achieve better screening results on highly flexible systems such as kinases.

**Table 7:** Screening success of the general scoring functions trained with MLR, SMOreg and RF evaluated on the AKT2, KIT, and MK01 datasets from DUD-E. *ac*, *dec* and *tot* are the number of active, decoy compounds and the total number of molecules in the final dataset (*i.e.,* compounds that were docked and rescored with DockThor and DockTScore, respectively). Only the top-scored protonation state of each compound according to each scoring function (SF) was kept.

Target	Metrics	General SFs		
		MLR	SMOreg	RF
AKT2	AUC	0.769	0.800	0.814
*ac* = 116	EF1% (max = 60.414)	24.166	15.535	13.809
*dec* = 6,892	BEDROC20	0.421	0.378	0.379
*tot* = 7,008	BEDROC100	0.394	0.288	0.269
KIT	AUC	0.640	0.635	0.657
*ac* = 166	EF1% (max = 63.934)	3.016	2.413	5.428
*dec* = 10,447	BEDROC20	0.148	0.146	0.176
*tot* = 10,613	BEDROC100	0.063	0.043	0.090
MK01	AUC	0.786	0.766	0.745
*ac* = 78	EF1% (max = 59.308)	10.314	12.893	7.736
*dec* = 4,548	BEDROC20	0.352	0.364	0.340
*tot* = 4.626	BEDROC100	0.153	0.220	0.193

## Discussion

**Figure 10:**
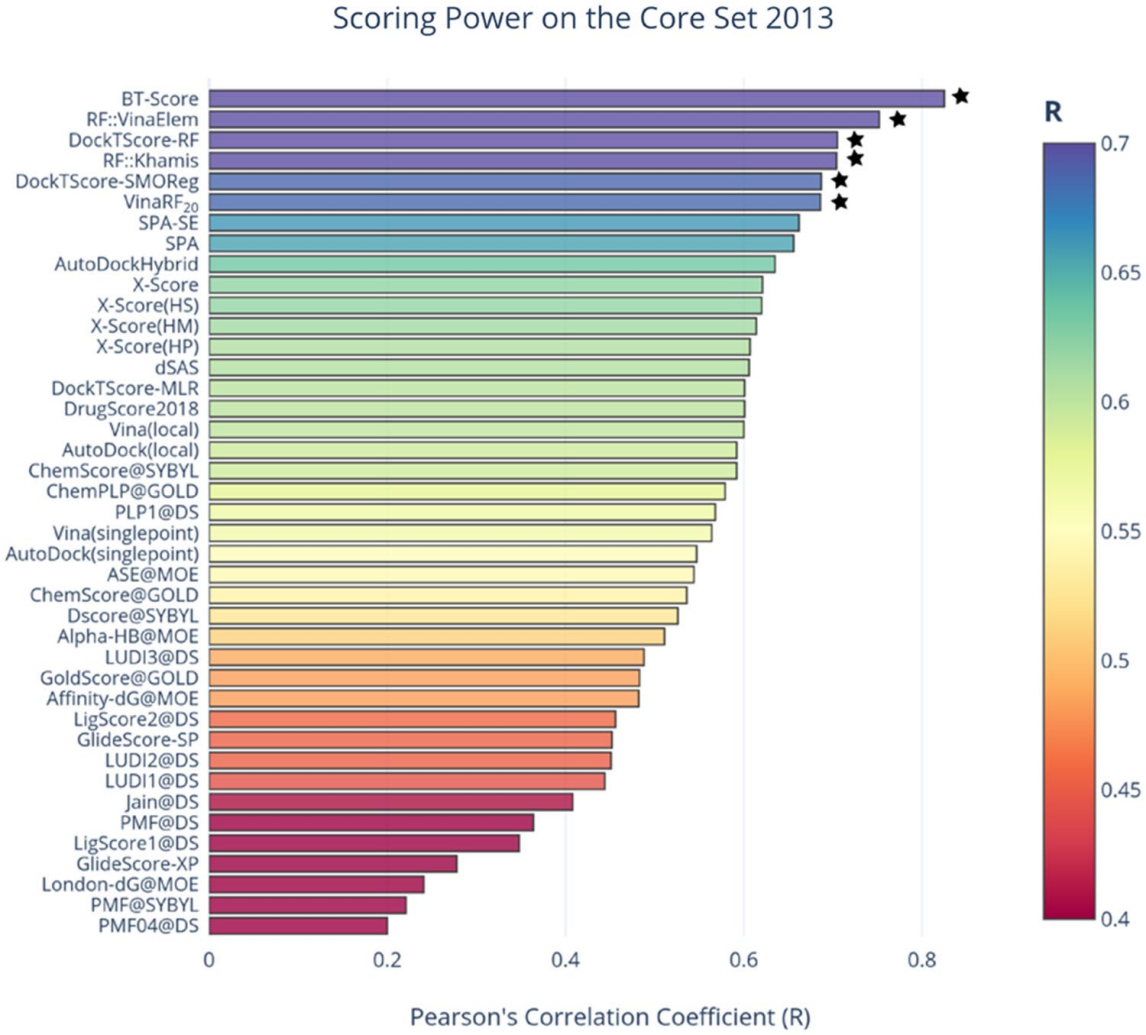
Scoring power of DockTScore linear and nonlinear models compared to the scoring functions evaluated on the core set 2013. Performances collected from the literature: BT-Score^75^, CompSPA^76^, AutoDockHybrid^23^, and the remaining were recalculated from raw data in the recent work of T. Gaillard^77^. Nonlinear models are highlighted with a star. Scoring functions with Pearson’s correlation coefficients higher or equal than 0.7 are colored purple and those lower than or equal to 0.4 are colored red.

We validated our physics-based terms for the general scoring functions using MLR. Despite its simplest form, MLR has the advantage to provide practical insights into relationships between the predicted binding affinity and the individual contribution of each specific term to the scoring function. In this work, all decisions regarding the selection of terms and machine-learning algorithms were made based on cross-validation experiments on the training set. The strategy of selecting random and independent test sets as employed here is particularly important in order to avoid performance overestimation. The performance of binding affinity prediction of the DockTScore general scoring functions are comparable with other well-known empirical scoring functions also tested on the v2013 PDBBind core set, *e.g.*, X-score::HMScore (R = 0.644)^57^, Surflex-Dock (R = 0.388)^73^, VinaRF_20_ (R = 0.686)^74^, and RF::VinaElem (R = 0.752) (Fig. 10). We obtained better performance on the carefully prepared core set compared to the random test set. One reason is that the selection of the complexes to form the *core set* ensured that all protein families in this benchmarking set were also present in the training set. Also, we believe that a correct preparation of the system, like the protonation state assignment as done for the *core set*, is important for proper binding energy prediction and a reliable assessment of scoring functions based on a more sophisticated protein–ligand interactions description.

Interestingly, the RF-Score::VinaElem (R = 0.752)^78^, a nonlinear scoring function based on 36 element-element distance counts, the five Vina scoring function energy terms and the number of rotatable bonds in the ligand, showed highest performance in comparison with other well-established scoring functions validated on the same v2013 core set^39^. On the other hand, it has been recently suggested that linear scoring functions, which can be less-accurate for binding affinity prediction but are composed of meaningful protein–ligand interaction terms, can be more robust than nonlinear scoring functions based only on element-element distance counts^4^. Definitely, element-element pair approaches are less sensitive to the proper dataset preparation, discarding the necessity of the time-consuming task of a careful assignment of the protonation states and atom types. However, scoring functions based on the calculation of physics-based binding energy terms might capture free energy changes arising from subtle protein–ligand interaction changes, useful particularly for hit-to-lead optimization.

It is widely recognized that target-specific scoring functions increase the efficiency of virtual screening exercises^21,24,27^. Different targeted docking-scoring strategies have been employed during the last decade. Some recent studies focused on combining scoring and pharmacophore/fingerprint filtering showed to improve target-specific pose/ligand selection^22,79,80^. We decided to develop new target-specific scoring functions for two protein classes to directly improve the prediction of the binding affinity by considering physics-based protein–ligand interaction terms. We obtained a remarkable improvement for the best nonlinear scoring function specific for PPIs (*i.e.,* the SMOReg model) compared to the general scoring function, achieving a significantly higher performance R = 0.613 against R = 0.431 obtained by the SMOReg general scoring function. For protease, such direct comparison is not reliable since most of the protease complexes present in the respective test set were also present in the training set used to derive the general scoring functions. Specific scoring functions have already been developed for well-established key protease targets as HIV-1 protease^28^ and their performances are comparable with our SMOreg models. The advantage of our targeted scoring functions for proteases compared to the above-cited studies is the physical interpretability of the terms describing the protein–ligand interactions and good performances on virtual screening experiments evaluated with AUC ROC, EF_1%_ and BEDROC metrics for the screening assessment.

Despite the insufficient accuracy exhibited by our linear scoring function specific for iPPIs on the independent test set, it served as a basis for the development of nonlinear models using SMOReg and RF techniques. As expected, the nonlinear scoring function specific for iPPIs, in particular SMOReg, showed a significant improvement of the predictive performance when compared with the MLR model in terms of binding affinity prediction. However, analyzing the virtual screening metrics for the Bcl2 target, we observe distinct results. The AUC values obtained using the SMOReg specific functions are better than the values obtained using MLR specific ones, yet the MLR specific function outperformed following the early recognition metrics (principally for EF1% and BEDROC 100). Thus, both the affinity prediction and ranking of compounds are important to properly evaluate the scoring functions performance. Our PPI-specific scoring functions were trained with 45 different PPI complexes covering thus a larger PPI interaction space than the previously used one for the only one reported linear scoring function specific for iPPIs HADDOCK2P2I^36^. The two PPI-specific scoring functions SMOReg and HADDOCK2P2I seem to perform similarly in terms of binding affinity prediction, yet the studies have been done on different PPI targets. To the best of our knowledge, the present SMOReg DockTScore is the first reported nonlinear scoring function tailored for the iPPI class that facilitates further optimization of the terms and the machine-learning algorithm used for training. In addition, the screening results obtained for the two PPI systems indicate that our PPI-specific scoring function trained with MLR is sufficiently robust to be used in virtual screening experiments, despite being trained with a small training set. Taking into consideration the very few scoring functions dedicated to score properly inhibitors of PPI both HADDOCK2P2I and DockTScore scoring functions can be very helpful e.g., for consensus scoring strategies. Furthermore, the growth of the number of experimentally derived iPPI structures available with associated affinity data enables the further development of more robust scoring functions specific for PPIs.

The variable performances achieved by the DockTScore models on the screening validation for the three different classes of proteins (*e.g.,* proteases, PPIs and kinases) are in agreement with other works published in the literature showing that the accuracy of scoring functions is strongly target-dependent. Further, although our scoring functions consider most of the interactions key for ligand binding, yet we do not take into account some contributions like the vibrational entropy^16^ or particular cases as water molecules present in the binding pocket. The vibrational entropy is strongly related to the protein flexibility and to solvent entropy, and their precise estimation is not evident to be included in classical scoring functions. Other approaches as molecular dynamics or normal mode analysis can help to resolve such problems, however they are unpractical for a huge number of ligands and thus they are out of the scope of this work. Kinases are known to be very flexible proteins, and in our study KIT is the kinase protein for which our models exhibited the lowest performances on both AUC ROC and early recognition capacity evaluated through EF1% and BEDROC. The protein conformation of KIT provided by the DUD-E database and used here as the reference structure is complexed with the kinase inhibitor sunitinib. That KIT state corresponds to a more closed conformation of the ATP-binding site. The superposition of the autoinhibited KIT complexed with sunitinib (PDB code 3G0E) and the KIT-ponatinib complex (PDB code 4U0I), ponatinib being larger than sunitinib, shows an induced inactive DFG-out conformation of the enzyme, illustrating thus two possible distinct conformations adopted by the enzyme due to different ligands (Figure S1). Such results reinforce the importance of a careful selection of the receptor conformation to be used for virtual screening campaigns and the consideration of the protein flexibility to some extent^81^.

Next, many inhibitors of proteases such as TRYB1 and UROK are known to displace water molecules interacting with catalytic residues of the binding site, however, in some cases such molecules can serve as a bridge between the receptor and the ligand. The analysis of the nine experimental complexes used in the virtual screening experiments showed that some of them contain ligands able to displace water molecules (*e.g.,* the proteases) and/or contain bridging waters in the experimental structure of the protein used in the virtual screening experiments (*e.g.,* FA7, TRYB1, and MDM2). In the case of MDM2-like protein, there is a complex network of water molecules mediating hydrogen bonds with the receptor important for the ligand binding. Such data support the importance of the enthalpic and entropic contributions of the water molecules in the binding pocket for the binding energy. The consideration of the contribution arising from bridged water molecules is a complex problem usually treated with more sophisticated methods that take into account the flexibility of the entire system and explicit water molecules. We have previously developed the AMMOS2 web server^82^, which permits to take into consideration the presence of explicit water molecules in the binding pocket in order to optimize the predicted protein–ligand interactions.

The better performance of our MLR scoring function specific for PPIs on the protease TRYB1 dataset indicates that it could be applied on targets with similar profiles with those observed for PPI interfaces, such as those with highly solvent-exposed binding sites. We have recently reported similar observations when analyzing solvent-exposed co-crystallized ligands to support the design of novel protein–protein interaction inhibitors^83^. Our scoring function specific for PPIs also reinforces the fact that nonlinear scoring functions are more dependent on larger training sets, while robust linear models can be developed even when scarce data for training is available. Future growth of data for new PPI interfaces including dimer interfaces will allow to develop more robust nonlinear scoring functions specific for protein targets with binding site profiles similar to those found in PPIs.

## Conclusion

In this work, we developed general and target-specific scoring functions using physics-based features for predicting binding affinities of protein–ligand complexes. Target-specific scoring functions were derived to account for binding characteristics specific for a target class of interest, focusing here on proteases and protein–protein interactions (PPIs). With regard to the increasing interest toward targeting PPIs by small-molecule inhibitors, here we reported the first and well-performing SVM-based scoring function specific for PPI binding sites that can serve as a valuable tool for discovering new iPPIs. Improved solvation and ligand torsional entropy terms were implemented in DockTScore for a reliable representation of ligand binding. DockTScore scoring functions demonstrated to be competitive with state-of-the-art scoring functions in reported benchmarking studies. As expected, the nonlinear scoring functions generally performed better than the respective MLR models. Finally, we demonstrated that the scoring functions developed in this work also exhibited good performances on virtual screening experiments to distinguish actives from inactive/decoy compounds for various protein targets. DockTScore functions are independent of docking software and can be used for affinity prediction or consensus scoring to improve the performance of docking-scoring approaches on virtual screening experiments. Currently, the MLR DockTScore predictions are provided for the DockThor docking at the DockThor-VS web server (available at www.dockthor.lncc.br). All the developed scoring functions in this work are under implementation in a dedicated web server.

## Supplementary Information

Supplementary Information.

## Data Availability

The curated PDBbind core set v2013, manually prepared to insure the correct protonation states of the protein–ligand complexes, is freely available at www.dockthor.lncc.br.
