# Supplementary material for: New machine learning and physics-based scoring functions for drug discovery
Source: Sci Rep. 2021 Feb 4;11:3198. doi: 10.1038/s41598-021-82410-1 (PMC7862620; doi:10.1038/s41598-021-82410-1)
Supplement: Supplementary file 1 — Supplementary Information. [file 41598_2021_82410_MOESM1_ESM.docx]

Supporting Information

**New machine learning and physics-based scoring functions for drug discovery**

*Isabella A. Guedes^1,3^, André M. S. Barreto^1^, Diogo Marinho^1^, Eduardo Krempser^2^, Mélaine A. Kuenemann^3^, Olivier Sperandio^3,4^, Laurent E. Dardenne^1^* and Maria A. Miteva^3,5^**

^1^ Laboratório Nacional de Computação Científica, Petrópolis 25651-075, Brazil;

^2^ Fundação Oswaldo Cruz, Rio de Janeiro 21040-361, Brazil;

^3^ Inserm U973, Université Paris Diderot, France;

^4^ Structural Bioinformatics Unit, CNRS UMR3528, Institut Pasteur, 75015 Paris, France;

^5^ Inserm U1268 “Medicinal Chemistry and Translational Research”, CiTCoM UMR 8038, CNRS, Université de Paris, Paris 75006, France.

* Corresponding authors

E-mail addresses: maria.mitev@inserm.fr, dardenne@lncc.br

Table S1. Composition of training and testing sets for general and target-specific scoring functions. The general scoring function was also evaluated using the test set v2013 core set as independent test set.

| **Target class** | **Source** | **Total** | **Training** | **Random Test Set** | **Core Set** |
| --- | --- | --- | --- | --- | --- |
| *General::random* | PDBbind v2013 | 2764 | 2073 | 691 | 195 |
| *General::all* | PDBbind v2013 | 2764 | 2764 | - | 195 |
| *Proteases* | PDBbind v2013 | 783 | 587 | 196 | - |
| *PPIs* | iPPI X-ray | 60 | 45 | 15 | - |

Table S2. Performances of the basic scoring functions composed of MMFF94S force-field terms trained with PDBbind v2013 refined set minus random test set and core set (N = 2073) evaluated in cross-validation experiments.

| van der Waals | Electrostatic | R_cross^a^ | RMSE^b^ |
| --- | --- | --- | --- |
| original Buf-14-7 | Coulomb $\varepsilon(r)\to1$ | 0.057 | 2.975 |
|  | Coulomb $\varepsilon(r)\to4$ | 0.068 | 2.936 |
| softened Buf-14-7 | Coulomb $\varepsilon(r)\to1$ | 0.491 | 2.349 |
|  | Coulomb $\varepsilon(r)\to4$ | 0.493 | 2.346 |
| ^a^ R is the Pearson correlation coefficient on 10-fold cross-validation (R_cross). ^b^ RMSE is the root mean squared error (in kcal mol^-1^). | | | |

Table S3. Performances of the linear scoring functions composed of the MMFF94S force-field terms *E_coul4_* and *E_vdWS_* and the lipophilic term trained with PDBbind v2013 refined set minus random test set and core set (N = 2073) evaluated in cross-validation experiments.

| Lipophilic term | Carbon atoms | | Partial charge^a^ | |
| --- | --- | --- | --- | --- |
|  | R_cross^b^ | RMSE^d^ | R_cross^b^ | RMSE^c^ |
| ChemScore | 0.519 | 2.306 | 0.538 | 2.274 |
| X-Score | 0.507 | 2.324 | 0.526 | 2.294 |
| ^a^ All atoms with a partial charge (q) in the interval -0.4 < q < +0.4. ^b^ R is the Pearson correlation coefficient on 10-fold cross-validation (R_cross). ^c^RMSE is the root mean squared error (in kcal mol^-1^). | | | | |

Table S4. Evaluation of the contribution of individual terms when associated with the basic scoring function composed of MMFF94S force-field terms (F_MMFF_), trained with PDBbind v2013 refined set minus random test set and core set (N = 2073).

| **Basic function** | **Feature** | **Variations** | **R^a^** | **RMSE^b^** |
| --- | --- | --- | --- | --- |
| F_MMFF_  R = 0.493  RMSE = 2.346 | *Lipophilic* | ChemScore functional form, nonpolar atoms defined by atomic number | 0.519 | 2.306 |
|  |  | ChemScore functional form, nonpolar atoms defined according to partial charges | 0.538 | 2.274 |
|  |  | X-Score functional form, nonpolar atoms defined by atomic number | 0.507 | 2.324 |
|  |  | X-Score functional form, nonpolar atoms defined according to partial charges | 0.526 | 2.294 |
|  | *Entropy* | Number of “frozen” rotatable bonds based on ΔSAS | 0.507 | 2.325 |
|  | *Polar solvation* | Number of charged atoms buried after binding and not interacting with a ligand charged atom. | 0.514 | 2.313 |
|  | *Nonpolar solvation* | Total loss of the solvent-accessible surface area (SAS) of the protein and the ligand  nonpolar atoms due to the binding. | 0.502 | 2.332 |

^a^ R is the Pearson correlation coefficient on 10-fold cross-validation (R_cross). ^b^ RMSE is the root mean squared error (in kcal/mol).

Table S5. Evaluated parameters of the SVM and RF algorithms.

| **Algorithm** | **Parameter** | **Values tested** | **N models** |
| --- | --- | --- | --- |
| *SMOReg* | *Complexity (C)* | {1, 2, …, 9, 10} | 3000 |
|  | *epsilon (ε)* | {0.00001, 0.0001, 0.001, 0.01, 0.1} |  |
|  | *Kernel* | *puk, rbf* |  |
|  | *puk: sigma (*σ*)* | {0.01, 0.05, 0.1, 0.5, 1} |  |
|  | *puk: omega (*ω*)* | {0.01, 0.05, 0.1, 0.5, 1} |  |
|  | *rbf: gamma (*γ*)* | {0.01, 0.05, 0.1, 0.5, 1} |  |
| *RF* | *numTrees* | {10, 15, 25, 50, 100, …, 450, 500} | 39 |
|  | *numFeatures* | {0, 1, 2, 3} |  |

Table S6. Optimal parameters of SVM for general and target-specific scoring functions.

| **Algorithm** | **Parameter** | **General** | **Protease** | **PPI** |
| --- | --- | --- | --- | --- |
| *SMOReg* | *Complexity* | 2 | 1 | 2 |
|  | *epsilon* | 0.00001 | 0.0001 | 0.0001 |
|  | *Kernel* | *puk* | *puk* | *puk* |
|  | *puk: sigma* | 0.1 | 1.0 | 1.0 |
|  | *puk: omega* | 0.1 | 0.1 | 0.1 |
| *RF* | *numTrees* | 200 | 100 | 15 |
|  | *numFeatures* | 2 | 3 | 3 |

Table S7. Performances of the linear and nonlinear scoring functions trained with MLR, SMOReg and RF. The target-specific scoring functions were validated on their respective independent test sets.

| **Algorithm** | **Scoring functions** | **R^a^ training** | **R cross** | **R test** | **RMSE^b^** |
| --- | --- | --- | --- | --- | --- |
| MLR | General::random | 0.553 | 0.548 | 0.602^c^ (0.494^d^) | 2.470^c^ (2.311^d^) |
|  | General::all | 0.539 | 0.536 | 0.601^c^ | 2.478^c^ |
|  | Proteases | 0.629 | 0.614 | 0.653 | 2.086 |
|  | iPPIs_oneSolv | 0.693 | 0.552 | 0.442 | 2.011 |
| SMOReg | General::random | 1.000 | 0.653 | 0.668^c^ (0.598)^d^ | 2.338^c^ (2.130^d^) |
|  | General::all | 1.000 | 0.647 | 0.687^c^ | 2.303^c^ |
|  | Proteases | 0.996 | 0.749 | 0.730 | 1.886 |
|  | iPPIs_oneSolv | 1.000 | 0.600 | 0.613 | 1.412 |
| RF | General::random | 0.975 | 0.655 | 0.678^c^ (0.613) ^d^ | 2.325^c^ (2.103)^d^ |
|  | General::all | 0.974 | 0.656 | 0.705^c^ | 2.256^c^ |
|  | Proteases | 0.974 | 0.735 | 0.723 | 1.901 |
|  | iPPIs_oneSolv | 0.946 | 0.666 | 0.478 | 1.813 |
| ^a^ R is the Pearson’s correlation coefficient on training set (R training), 10-fold cross-validation (R cross), test set (R test). ^b^ RMSE is the root mean squared error (in kcal mol^-1^). ^c^ Performance on the curated core set (N = 195). ^d^ Performance on the random test set (N = 691). | | | | | |


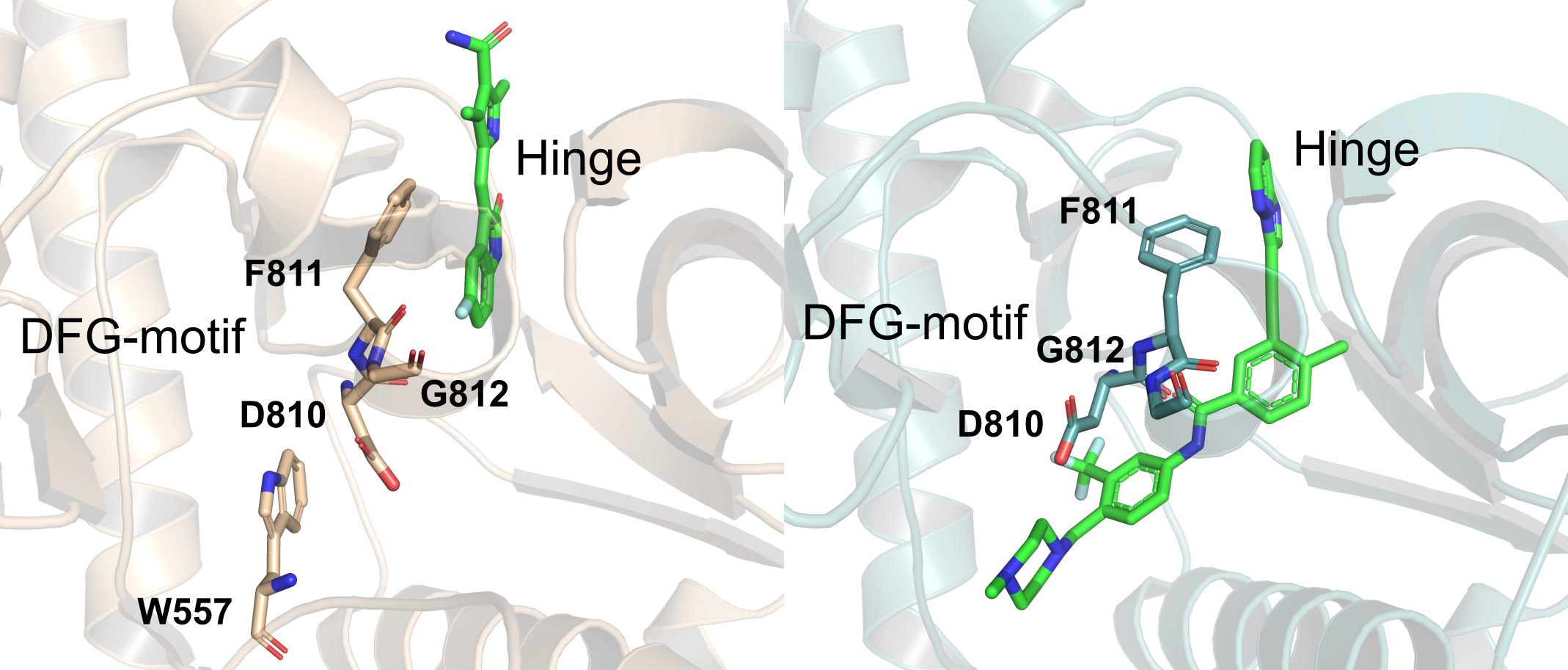
Figure S1. Experimental structures of KIT autoinhibited complexed with sunitinib (PDB code 3G0E, left) and ponatinib complex (PDB code 4U0I), which is larger than sunitinib and induces the inactive DFG-out conformation of the enzyme (right). The co-crystallized ligands are represented as green carbon sticks. The key regions for interaction of the most potent inhibitors, hinge and DFG-motif, are highlighted in the picture.
